# Genetic analysis of pyrimidine biosynthetic enzymes in *Plasmodium falciparum*

**DOI:** 10.1371/journal.ppat.1014269

**Published:** 2026-05-27

**Authors:** Krithika Rajaram, Montana L. Sievert, Rubayet Elahi, James Blauwkamp, Lucas B. Dillard, Sabrina Absalon, Sean T. Prigge

**Affiliations:** 1 Department of Molecular Microbiology and Immunology, Johns Hopkins Bloomberg School of Public Health, Baltimore, Maryland, United States of America; 2 Department of Microbiology, The Ohio State University, Columbus, Ohio, United States of America; 3 Department of Biochemistry, Molecular Biology and Toxicology, Indiana University School of Medicine, Indianapolis, Indiana, United States of America; 4 Department of Biophysics and Biophysical Chemistry, Johns Hopkins University School of Medicine, Baltimore, Maryland, United States of America; University of Geneva Faculty of Medicine: Universite de Geneve Faculte de Medecine, SWITZERLAND

## Abstract

The malaria parasite *Plasmodium falciparum* depends entirely on *de novo* pyrimidine synthesis, as it is unable to salvage these essential nucleotides. This reliance makes the pyrimidine biosynthesis pathway a compelling target for antimalarial drugs, with several inhibitors targeting its rate-limiting enzyme, dihydroorotate dehydrogenase (*Pf*DHODH), already in clinical development. In this study, we investigated the roles of three other pathway enzymes: aspartate transcarbamoylase (*Pf*ATC), carbamoyl phosphate synthetase II (*Pf*CPSII), and dihydroorotase (*Pf*DHO). *Pf*ATC features a unique N-terminal extension predicted to serve as an apicoplast trafficking peptide. However, using antibodies against the native protein and epitope-tagged versions, we found no evidence of apicoplast localization. Knockdown of *Pf*ATC expression proved lethal and could not be rescued by an apicoplast metabolic bypass. Complementation assays further revealed that truncation of the N-terminal domain impaired parasite growth, suggesting that this region is important for *Pf*ATC function or stability *in vivo*. *Pf*CPSII, which harbors large *Plasmodium*-specific insertions between its catalytic domains, was likewise found to be essential for parasite proliferation. To assess the role of *Pf*DHO, we engineered parasites to salvage uracil via heterologous expression of a yeast enzyme. Deletion of *Pf*DHO in this parasite line resulted in uracil auxotrophy, confirming the enzyme’s essential function in pyrimidine synthesis. Together, these findings reveal multiple vulnerable nodes within the pyrimidine biosynthesis pathway.

## Author summary

Nucleotides are central metabolites that serve as building blocks for DNA and RNA, act as key energy carriers, and function as cofactors or regulators in several metabolic pathways. To satisfy these diverse demands, most organisms rely on both nucleotide salvage and *de novo* synthesis. The malaria parasite *Plasmodium falciparum* acquires purine nucleotides from the host but lacks the capacity to salvage pyrimidines, making *de novo* pyrimidine synthesis essential. Several enzymes in this pathway differ from their human counterparts in sequence, domain architecture, and evolutionary origin, enhancing their potential as selective drug targets. Dihydroorotate dehydrogenase (PfDHODH), the fourth enzyme in the pathway, has already been validated as an antimalarial target. Here, we systematically examined upstream enzymes using molecular genetic approaches. Each proved essential for asexual blood-stage parasite survival, with the *Plasmodium*-specific N-terminal extension of aspartate carbamoyltransferase (*Pf*ATC) required for optimal growth. The introduction of a yeast uracil salvage enzyme rescued parasites depleted of these biosynthetic enzymes, demonstrating that their essential functions are confined to pyrimidine production and that their distinctive structural features do not support additional metabolic roles. In summary, these results delineate additional enzymatic steps in this important metabolic pathway that warrant continued investigation from both biological and translational angles.

## Introduction

The transition from a free-living to an obligate parasitic lifestyle is marked by a reduction of core metabolic pathways and the acquisition of nutrient salvage mechanisms, leading to irreversible dependence on the host. Apicomplexan parasites, which cause a range of human and animal diseases, have undergone genomic streamlining to adapt to their host niches, enabling them to salvage key building blocks like amino acids, lipids, and nucleotides [1–3]. While all apicomplexans rely on purine salvage, their capacity for pyrimidine uptake varies [4–6]. *Plasmodium falciparum*, the causative agent of human malaria, is unable to use exogenous pyrimidines and requires *de novo* synthesis, making this pathway an attractive target for therapeutic intervention [7–11].

**Fig 1:**
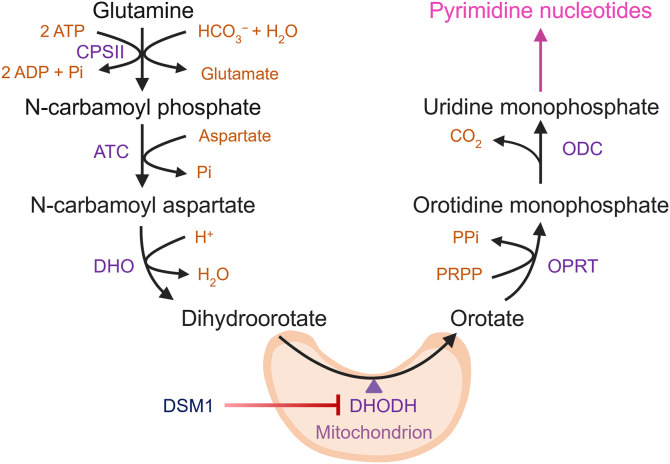
Pyrimidine biosynthesis in *P. falciparum.* This figure illustrates the enzymatic reactions involved in the *de novo* biosynthesis of pyrimidines in *P. falciparum*. The pathway begins with the conversion of glutamine and bicarbonate into carbamoyl phosphate by the enzyme carbamoyl phosphate synthetase II (*Pf*CPSII). Subsequent steps catalyzed by aspartate transcarbamoylase (*Pf*ATC) and dihydroorotase (*Pf*DHO) lead to the formation of dihydroorotate. Dihydroorotate is then oxidized to orotate by dihydroorotate dehydrogenase (*Pf*DHODH), a mitochondrial enzyme that can be inhibited by a class of triazolopyrimidine compounds including DSM1 [13]. Orotate is subsequently converted to orotidine-5’-monophosphate (OMP) by orotate phosphoribosyltransferase (*Pf*OPRT), and OMP is decarboxylated to uridine 5’-monophosphate (UMP) by orotidine-5’-monophosphate decarboxylase (*Pf*ODC). UMP is a precursor for the synthesis of all pyrimidine nucleotides, which are essential for DNA and RNA synthesis.

Clinical symptoms of malaria stem from the rapid proliferation of *P. falciparum* within host red blood cells, which, being anucleate, have low nucleotide demands and limited pyrimidine availability [12]. The parasite sustains its high metabolic needs by synthesizing the pyrimidine uridine 5’-monophosphate (UMP) through a conserved six-step pathway that begins with carbamoyl phosphate synthetase II (CPSII) (Fig 1) [14,15]. CPSII catalyzes the formation of carbamoyl phosphate from bicarbonate and glutamine [16,17]. Carbamoyl phosphate then undergoes condensation with aspartate in a reaction catalyzed by aspartate transcarbamoylase (ATC) to yield carbamoyl aspartate and inorganic phosphate [18–20].

Dihydroorotase (DHO) catalyzes the next reaction in the pathway, converting carbamoyl aspartate to dihydroorotate [21,22]. Dihydroorotate is oxidized to orotate by dihydroorotate dehydrogenase (DHODH), a flavin-dependent mitochondrial enzyme that uses ubiquinone as a terminal electron acceptor in malaria parasites [23]. *P. falciparum* DHODH (*Pf*DHODH) activity is thought to be the primary driver for maintaining mitochondrial electron transport chain (mETC) function in asexual blood-stage parasites, which primarily rely on glycolysis for ATP production [24–26]. The role of the mETC during this stage appears to be largely limited to the regeneration of ubiquinone for *Pf*DHODH [24]. Consequently, mETC inhibitors like atovaquone are ineffective against *P. falciparum* lines engineered to express a cytosolic yeast DHODH homolog that uses fumarate instead of ubiquinone [24,27–30].

Orotate phosphoribosyltransferase (OPRT) performs the penultimate step in the pathway, transferring a ribose 5-phosphate group from phosphoribosyl pyrophosphate to orotate to form orotidine 5′-monophosphate (OMP) [23,31,32]. OMP is then converted to the pathway product UMP by OMP decarboxylase (ODC) [23,32]. UMP is subsequently converted into other pyrimidine and deoxypyrimidine nucleotides through a series of enzymatic steps [7,14].

**Fig 2:**
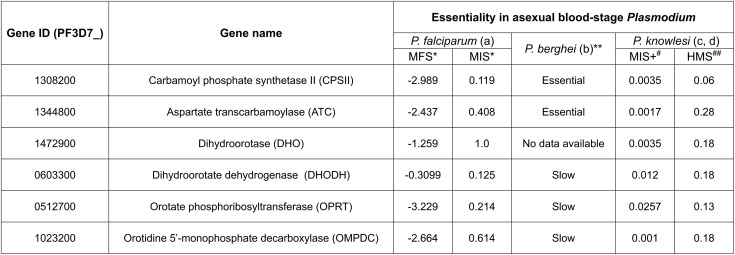
Essentiality of pyrimidine biosynthesis pathway genes from genetic screens in *Plasmodium*. a – Zhang et al., 2018 [41]; b – Schwach et al., 2015 [42]; c – Oberstaller et al., 2025 [43]; d – Elsworth et al., 2025 [44]. * The genome-wide screen for essential genes in *P. falciparum* employed the mutagenesis fitness score (MFS; ranging from -4.5 to 2) and mutagenesis index score (MIS; ranging from 0 to 1), to assess gene mutability [41]. Low MFS and MIS scores are indicative of gene essentiality. ** The PlasmoGEM knockout screen in the murine malaria parasite *P. berghei* used relative growth rates to assign growth phenotypes [42]. Genes were classified as dispensable if the 95% confidence interval included a relative growth rate of 1.0, essential if it included 0.1, slow if it ranged between 0.1 and 1.0, and fast if it exceeded 1.0. ^#^ A genome-wide screen for essential genes in *P. knowlesi* incorporated the mutagenesis index score + (MIS+ ; ranging from 0 to 1) to assess gene mutability [43]. Low MIS+ scores are indicative of gene essentiality. ^##^ A genome-wide screen for essential genes in *P. knowlesi* incorporated the hybrid model score (HMS) to predict gene essentiality. An HMS score below 0.26 indicates essentiality, whereas a score above 0.88 indicates that the gene is dispensable [44].

Phylogenetic analyses reveal that the pyrimidine biosynthetic enzymes in *P. falciparum* have mixed evolutionary origins: *Pf*CPSII, *Pf*DHO, and *Pf*OPRT are more closely related to prokaryotic orthologs, while the remaining enzymes exhibit features of both bacterial and eukaryotic lineages [11,14]. *Pf*CPSII and *Pf*ATC are also unusual in that they contain large insertions of unknown function [15,20,33]. Beyond these sequence-level distinctions, the structural organization of the pathway also differs markedly from that of the human host [11,14]. In humans and other multicellular eukaryotes, the first three steps of pyrimidine biosynthesis are catalyzed by a single multifunctional protein, CAD, which combines the activities of CPSII, ATC, and DHO [34]. Similarly, OPRT and ODC are fused into a bifunctional enzyme known as UMP synthase in many eukaryotes [32,35]. In *P. falciparum*, however, *Pf*OPRT and *Pf*ODC are expressed as separate proteins that form a heterotetrameric complex [23,36–40]. The distinct architecture and evolutionary divergence of the *Plasmodium* enzymes from their human counterparts further enhance their potential as selective drug targets. Consistent with this, gene essentiality screens in *P. falciparum* and *P. berghei* indicate that most of these enzymes are required for parasite survival or associated with significant fitness costs (Fig 2) [41]. Notably, *Pf*DHODH, the only clinically validated target in this pathway, has already been the focus of extensive antimalarial drug development efforts [13,45–55].

In this study, we systematically dissected the pyrimidine biosynthetic pathway in *P. falciparum*, using gene knockdown and metabolic bypass approaches to investigate the roles of multiple enzymes. While *Pf*CPSII and *Pf*ATC possess unique sequence features absent from host counterparts, and DHODH has been reported to perform an additional function in *Toxoplasma gondii* [56], our findings demonstrate that these enzymes, along with *Pf*DHO, play a singular, essential role in pyrimidine synthesis during the asexual blood stage. By defining the essential metabolic function of these parasite enzymes, our work expands the pool of candidate antimalarial targets within this important biosynthetic pathway.

## Results

### P. *falciparum* aspartate transcarbamoylase (*Pf*ATC) is not an apicoplast-resident protein

**Fig 3:**
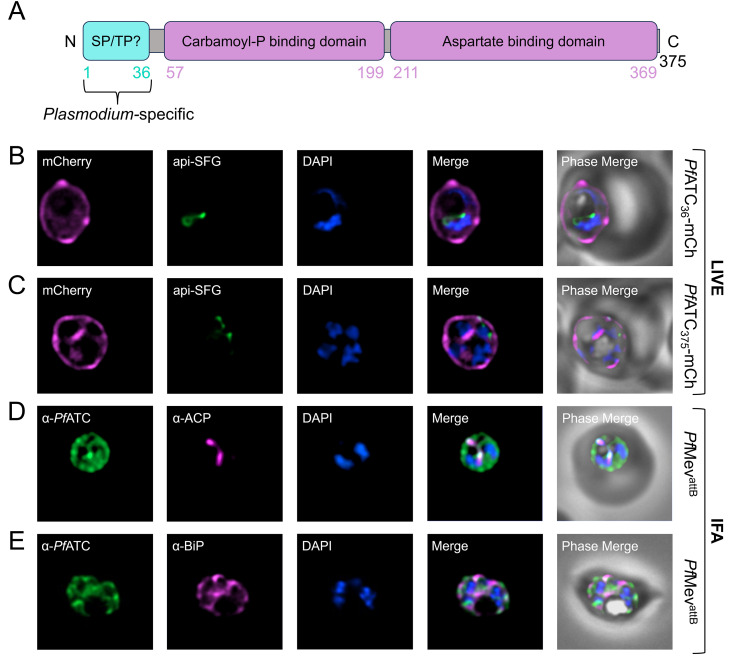
*Pf*ATC does not localize to the apicoplast. **(A)** Predicted (cyan) and annotated (purple) functional domains of the *Pf*ATC protein are depicted. The first 36 amino acids of *Pf*ATC are predicted to form a signal peptide (SP) and an apicoplast transit peptide (TP). **(B-C)** Representative live fluorescence images of parasites expressing **(B)** truncated or **(C)** full-length *Pf*ATC fused to mCherry (mCh) showed that the tagged proteins are mostly peripherally located and do not colocalize with the apicoplast marker api-SFG. **(D-E)** Representative images from immunofluorescence assays (IFA) on parental *Pf*Mev^attB^ parasites probed with α-*Pf*ATC and **(D)** apicoplast (α-ACP), or **(E)** ER-specific (α-BiP) antibodies did not reveal substantial overlap of signals. Quantification by Manders’ coefficient M1 (green signal within magenta signal) yielded a value of 0.138 (±0.098; n = 19) for α-*Pf*ATC and α-ACP, and 0.372 (±0.221; n = 19) for α-*Pf*ATC and α-BiP. DAPI (blue) stains the parasite nucleus. Images represent fields that are 10 μm long by 10 μm wide.

The ATC gene in *P. falciparum* encodes a 375-amino acid (aa) protein (*Pf*ATC), which has been demonstrated by previous structural and biochemical analyses to form a homotrimer [20,57]. Unlike its *E. coli* counterpart, the functional *Pf*ATC complex lacks regulatory subunits, suggesting it is not subject to feedback inhibition by the pathway’s end-product, UMP [19,20,33]. *Pf*ATC features an N-terminal extension that is conserved among *Plasmodium* species but is absent in orthologs from animals, fungi, and bacteria (Fig 3A). This domain is predicted to function as an apicoplast trafficking peptide based on analyses from three different bioinformatic tools: PATS, ApicoAP, and PlasmoAP [58–60]. A previous attempt to determine the subcellular location of *Pf*ATC showed that a GFP-tagged copy of the protein localized to a region distinct from the endoplasmic reticulum (ER); however, since the study did not include an apicoplast-specific marker, the precise location of *Pf*ATC remains unresolved [20].

To determine if *Pf*ATC is indeed an apicoplast protein, we fused a C-terminal mCherry tag to the first 36-aa of *Pf*ATC (*Pf*ATC_36_-mCh) which corresponds to the predicted apicoplast trafficking peptide. The fusion protein was expressed from a plasmid integrated into an *att*B site in *Pf*Mev^attB^ parasites (S1A, S1B and S1C Fig) [61,62]. This parasite line features a built-in fluorescent apicoplast reporter consisting of the apicoplast trafficking peptide of acyl carrier protein fused to superfolder GFP (api-SFG). Live microscopy revealed that *Pf*ATC_36_-mCh localizes primarily to the parasite periphery, along with a weaker cytoplasmic signal that does not overlap with the apicoplast (Fig 3B). Expression of full-length ATC fused to mCherry (*Pf*ATC_375_-mCh) also displayed a similar localization pattern, showing no overlap with api-SFG (Figs 3C and S1D).

To assess the location of endogenous *Pf*ATC, we generated antibodies against recombinant *Pf*ATC protein (S2 Fig). Immunofluorescence imaging of parasites using the α-*Pf*ATC antibodies revealed predominant staining in the parasite cytoplasm, with some peripheral signal (Fig 3D and 3E). Co-labeling with antibodies against apicoplast (ΑCP) or ER (BIP) markers showed minimal overlap, as reflected by low Manders coefficients: 0.138 ± 0.098 (n = 19) for α-PfATC with α-ACP, and 0.372 ± 0.221 (n = 19) for α-PfATC with α-BiP (Fig 3D and 3E). Overall, our findings indicate that *Pf*ATC is not located in the apicoplast, but it may be present in more than one subcellular location.

### *Pf*ATC is essential in blood-stage malaria parasites

Given its role in pyrimidine synthesis, we anticipated that blocking *Pf*ATC expression would be lethal to blood-stage malaria parasites. Consistent with this, *Pf*ATC was assigned a high essentiality score in a forward genetic screen in *P. falciparum* (Fig 2) [41]. For further validation, we employed a TetR-aptamer system to conditionally knock down *Pf*ATC expression [63,64]. A linearized pKD plasmid carrying TetR-DOZI components was introduced at the 3’ end of the *Pf*ATC gene in *Pf*Mev^attB^ parasites using CRISPR/Cas9-mediated gene editing (S3A and S3B Fig) [65]. The modified *Pf*ATC gene encodes a C-terminal 2× FLAG tag for protein visualization, and a 10× aptamer array in its 3’ UTR so the transcribed mRNA can be regulated by the TetR-DOZI module and its ligand anhydrotetracycline (aTet). The genotype of the conditional knockdown (*Pf*ATC^CD^) parasite line was confirmed by PCR (S3C Fig). The parasites were maintained continuously in medium containing aTet to allow translation of *Pf*ATC.

**Fig 4:**
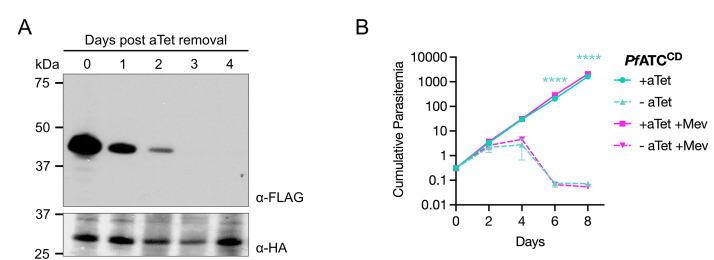
*Pf*ATC is essential for the survival of blood-stage *P. falciparum* parasites. **(A)** Western blot analysis using an α-FLAG antibody to probe for *Pf*ATC (expected MW: 45 kDa) revealed efficient knockdown of expression following aTet removal from *Pf*ATC^CD^ parasite cultures. An α-hemagglutinin (HA) antibody was used to detect HA-tagged api-SFG (expected MW of processed form: 28.1 kDa), which served as a loading control. **(B)** Growth of *Pf*ATC^CD^ parasites in the presence or the absence of aTet and mevalonate (Mev) was monitored by flow cytometry for 8 days. Removal of aTet led to parasite death, which could not be rescued by Mev addition. Two independent biological experiments were performed in quadruplicate. The means of technical replicates from each experiment were used for plotting and statistical analysis using GraphPad Prism 10 (GraphPad Software, Inc). Cumulative parasitemia was plotted on a log-scale Y-axis with standard deviation. Data were analyzed using two‑way ANOVA, followed by Bonferroni’s multiple‑comparison correction (****, P ≤ 0.0001; significant differences are shown for pairwise comparisons of the -aTet and -aTet +Mev conditions relative to the +aTet control).

To determine if *Pf*ATC is an essential protein, parasites were washed free of aTet to knock down *Pf*ATC expression, and their growth was monitored for 8 days. Immunoblotting with an α-FLAG antibody revealed strong expression of FLAG-tagged *Pf*ATC in parasite lysates on Day 0, which gradually decreased to undetectable levels by Day 3 (Fig 4A). By Day 4, parasites cultured without aTet displayed a noticeable growth defect (Fig 4B).

Parasites with apicoplast defects in the *Pf*Mev^attB^ background can be rescued by the addition of mevalonate, which is converted into essential isoprenoid building blocks through an engineered cytosolic pathway [61]. To determine if an apicoplast bypass could partially or fully rescue *Pf*ATC-depleted parasites, a fraction of the aTet-free *Pf*ATC^CD^ culture was split into media containing mevalonate on Day 0 of the growth assay. The addition of mevalonate did not alter the kinetics of parasite death (Fig 4B). From these data, we conclude that *Pf*ATC is an essential protein whose function cannot be complemented by a metabolic bypass for the apicoplast. This finding, together with the observed localization pattern for *Pf*ATC (Fig 3), suggests that the enzyme plays an essential role outside of the apicoplast.

### N-terminal truncation of *Pf*ATC impacts parasite growth

**Fig 5:**
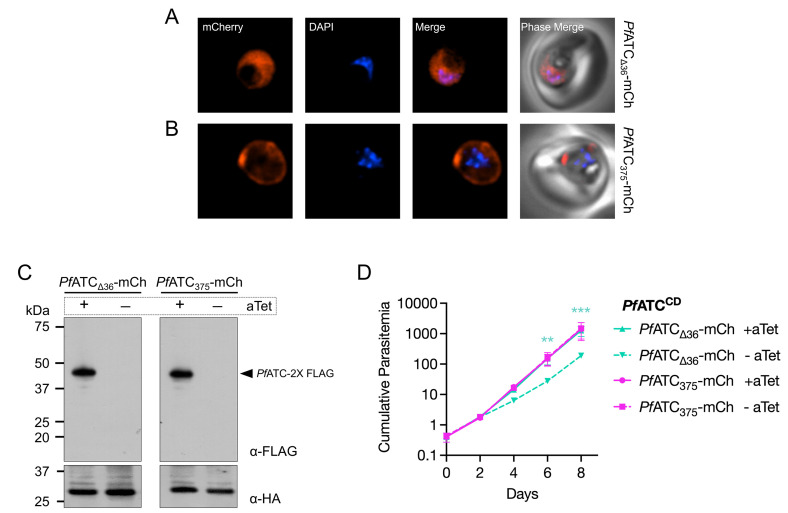
N-terminal truncation of *Pf*ATC impacts its localization and parasite growth. Representative live fluorescence microscopy images of *Pf*ATC^CD^ parasites expressing **(A)** truncated (*Pf*ATC_Δ36_-mCh) or **(B)** full-length (*Pf*ATC_375_-mCh) constructs of *Pf*ATC fused to mCherry showed that N-terminal truncation resulted in a shift from peripheral localization to diffuse cytoplasmic distribution of *Pf*ATC. Images represent fields that are 10 μm long by 10 μm wide. **(C)** Western blot analysis using an α-FLAG antibody on parasite lysates collected from Day 0 (+) and Day 8 (-) of aTet washout revealed efficient knockdown of endogenous FLAG-tagged *Pf*ATC (45 kDa). The α-HA antibody detecting HA-tagged api-SFG (expected MW of processed form: 28.1 kDa) was used as a loading control. **(D)** Growth of *Pf*ATC^CD^ parasites in the absence of aTet was entirely rescued by second-copy expression of *Pf*ATC_375_-mCh, and only partially rescued by *Pf*ATC_Δ36_-mCh. Two independent biological experiments were performed in quadruplicate. The means of technical replicates from each experiment were used for plotting and statistical analysis using GraphPad Prism 10 (GraphPad Software, Inc). Cumulative parasitemia was plotted on a log-scale Y-axis with standard deviation. Data were analyzed using two‑way ANOVA, followed by Bonferroni’s multiple‑comparison correction (**, P ≤ 0.01; ***, P ≤ 0.001; asterisks indicate significant differences between +aTet and -aTet *Pf*ATC_Δ36_-mCh conditions).

Since the N-terminal extension of *Pf*ATC is an uncommon feature for aspartate transcarbamoylases (Fig 3A), we asked if its removal would have any impact on the protein’s function in *P. falciparum* parasites. An mCherry tag was fused to the C-terminus of truncated *Pf*ATC missing the first 36-aa (*Pf*ATC_Δ36_-mCh). The fusion protein was expressed from a chromosomally integrated plasmid in the *Pf*ATC^CD^ parasite line (S4A Fig). A control parasite line was generated by introducing a second copy of full-length *Pf*ATC (*Pf*ATC_375_-mCh) into *Pf*ATC^CD^ parasites (S4B Fig). Live microscopy of *Pf*ATC_Δ36_-mCh parasites revealed diffuse mCherry signal throughout the parasite cytoplasm as opposed to the peripheral signal observed with *Pf*ATC_375_-mCh (Fig 5A and 5B). These results demonstrate that the N-terminus of *Pf*ATC affects protein localization.

Immunofluorescence assays using α-mCherry and α-FLAG antibodies similarly showed a more pronounced peripheral localization of *Pf*ATC_375_-mCh, with additional cytoplasmic signal (S5 Fig). By contrast, the endogenous FLAG-tagged *Pf*ATC displayed a more diffuse pattern with some peripheral distribution, phenocopying the localization observed with the α-*Pf*ATC antibody generated in this study (Figs S5, 3D and 3E). This suggests that expression of full-length *Pf*ATC from a strong non-native promoter enhances its peripheral distribution.

Next, we examined if truncated *Pf*ATC could rescue parasites depleted of endogenous *Pf*ATC. The *Pf*ATC^CD^ parasites expressing truncated or full-length *Pf*ATC-mCh were cultured in media with or without aTet, and their growth was monitored over a period of 8 days. Successful knockdown of endogenous *Pf*ATC was confirmed in cultures lacking aTet (Fig 5C). Parasites expressing *Pf*ATC_375_-mCh grew normally even without aTet, demonstrating that the full-length fusion protein can fully complement native *Pf*ATC function (Fig 5D). By contrast, expression of *Pf*ATC_Δ36_-mCh only partially rescued growth, with nearly a 10-fold reduction in cumulative parasitemia on days 6 and 8 of the growth assay (Fig 5D). Taken together, our results show that removing the N-terminus of *Pf*ATC impacts its subcellular location and parasite fitness.

### *Pf*CPSII and *Pf*DHO are required for parasite survival

**Fig 6:**
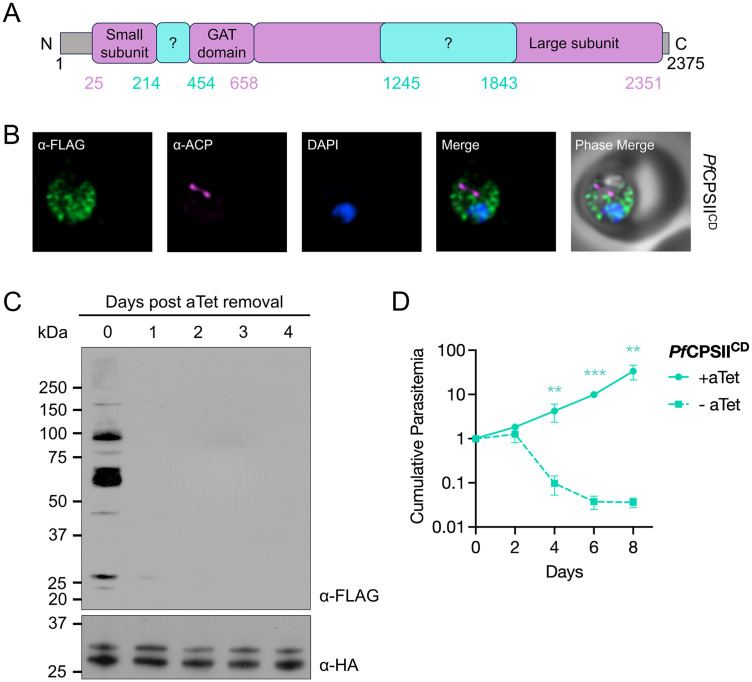
*Pf*CPSII is an essential protein in blood-stage *P. falciparum* parasites. **(A)** Insertions (cyan) and annotated (purple) functional domains of the *Pf*CPSII protein are depicted. GAT – glutamine amidotransferase domain. **(B)** Representative images from immunofluorescence assays (IFA) on *Pf*CPSII^CD^ parasites probed with α-FLAG and α-ACP (apicoplast) antibodies indicated that endogenous FLAG-tagged CPSII localized to the parasite cytoplasm. Images represent fields that are 10 μm long by 10 μm wide. **(C)** Western blot analysis using an α-FLAG antibody to probe for *Pf*CPSII revealed efficient knockdown of expression following aTet removal from *Pf*CPSII^CD^ parasite cultures. The α-HA antibody detecting HA-tagged api-SFG was used as a loading control. The two bands indicate precursor (33 kDa) and mature (28.1 kDa) forms of the apicoplast protein. **(D)** Growth of *Pf*CPSII^CD^ parasites in the presence or the absence of aTet was measured by flow cytometry for 8 days. Removal of aTet led to parasite death. Two biological experiments were conducted in quadruplicate. The means of technical replicates from each experiment were used for plotting and statistical analysis using GraphPad Prism 10 (GraphPad Software, Inc). Cumulative parasitemia was plotted on a log-scale Y-axis with standard deviation. Data were analyzed using two‑way ANOVA, followed by Bonferroni’s multiple‑comparison correction (**, P ≤ 0.01; ***, P ≤ 0.001).

The substrate for ATC, carbamoyl phosphate, is synthesized by carbamoyl phosphate synthetase II (CPSII). ATC then converts it into carbamoyl aspartate, which is subsequently utilized by dihydroorotase (DHO) (Fig 1). The *P. falciparum* CPSII (*Pf*CPSII) gene encodes a 2375-aa product with long insertions between putative functional domains, making it one of the largest known CPS family enzymes (Fig 6A) [15]. *Pf*CPSII was predicted to be essential in the forward genetic screen, whereas *Pf*DHO was unexpectedly deemed dispensable (Fig 2) [41]. Since neither gene has been characterized in depth, we generated conditional knockdown lines for *Pf*CPSII (*Pf*CPSII^CD^) and *Pf*DHO (*Pf*DHO^CD^) in the *Pf*Mev^attB^ background to determine their essentiality in blood-stage malaria parasites (S6A–C Fig). As before, the endogenous genes were modified to encode C-terminal FLAG tags for tracking protein fate. Immunofluorescence microscopy indicated that *Pf*CPSII-2× FLAG localizes to the cytoplasm (Fig 6B). Unfortunately, *Pf*DHO could not be reliably detected.

The transgenic parasites were divided into media with and without aTet and monitored for 8 days. The levels of *Pf*CPSII in the *Pf*CPSII^CD^ parasites were followed by immunoblotting with an α-FLAG antibody. While the expected size of *Pf*CPSII is ~ 273 kDa, we did not detect a protein at this molecular weight. Instead, we observed several smaller bands on Day 0 that were no longer detectable in parasites after aTet removal, suggesting that they are processed or degradation products of *Pf*CPSII (Fig 6C). The aTet-depleted *Pf*CPSII^CD^ parasites exhibited a pronounced growth defect by Day 4, confirming that *Pf*CPSII is required for blood-stage development of *P. falciparum* (Fig 6D).

The *Pf*DHO^CD^ parasites did not show appreciable reduction in *Pf*DHO protein levels upon aTet removal and displayed only modest fitness defects under these conditions (S6D and S6E Fig). Since the knockdown was not very effective, we attempted to knock out the *Pf*DHO gene in *Pf*Mev^attB^ parasites using CRISPR/Cas9. Despite multiple attempts, we failed to generate a deletion line, suggesting that *Pf*DHO may be essential.

**Fig 7:**
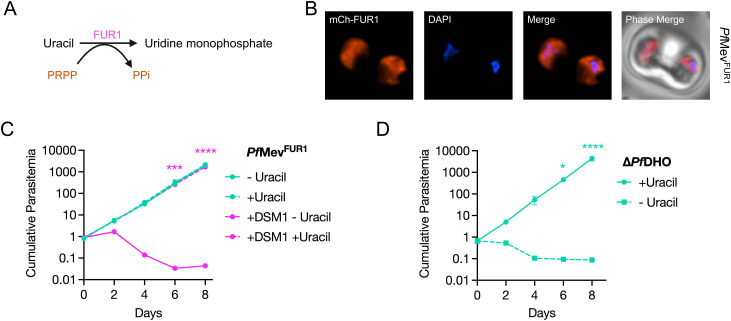
Introduction of a pyrimidine salvage enzyme in *P. falciparum* demonstrates that *Pf*DHO is essential for blood-stage growth. **(A)** The yeast enzyme FUR1 (uracil phosphoribosyltransferase) transfers a phosphoribosyl group from 5-phosphoribosyl-α-1-pyrophosphate (PRPP) to uracil to yield uridine 5’-monophosphate (UMP), the same product generated by the pyrimidine biosynthetic pathway. **(B)** Live fluorescence microscopy confirmed the expression of the mCherry-tagged FUR1 (mCh-FUR1) protein in *Pf*Mev^FUR1^ parasites. Images represent fields that are 10 μm long by 10 μm wide. **(C)** Growth of *Pf*Mev^FUR1^ parasites in the presence or the absence of uracil and DSM1 was measured by flow cytometry for 8 days. Growth inhibition from DSM1 treatment was averted when *Pf*Mev^FUR1^ parasites were supplemented with uracil. **(D)** Growth measurements of the Δ*Pf*DHO line across an 8-day period showed that the parasites relied on uracil salvage for survival. Removal of uracil from the culture medium led to rapid parasite death. Growth curves in **(C)** and **(D)** were generated from two biological experiments conducted in quadruplicate. The means of technical replicates from each experiment were used for plotting and statistical analysis using GraphPad Prism 10 (GraphPad Software, Inc). Cumulative parasitemia was plotted on a log-scale Y-axis with standard deviation. Data were analyzed using two‑way ANOVA, followed by Bonferroni’s multiple‑comparison correction (*, P ≤ 0.05; ***, P ≤ 0.001; ****, P ≤ 0.0001). Asterisks in **(C)** depict significant differences between +DSM1 -Uracil and +DSM1 +Uracil conditions.

To further investigate gene essentiality, we repeated the *Pf*DHO deletion experiments in the presence of a controllable pyrimidine bypass system. We introduced the *Saccharomyces cerevisiae* FUR1 gene (uracil phosphoribosyltransferase), tagged at the N-terminus with mCherry (mCh-FUR1), into the *att*B locus of *Pf*Mev^attB^ parasites to create the *Pf*Mev^FUR1^ line (S7A Fig) [66,67]. FUR1 is expected to convert supplemented uracil into UMP, the end-product of *de novo* pyrimidine biosynthesis (Fig 7A). Live microscopy confirmed the expression of mCh-FUR1 in the parasite cytoplasm (Fig 7B). To test the functionality of FUR1, we asked whether uracil supplementation could rescue *Pf*Mev^FUR1^ parasites from growth inhibition caused by DSM1, a drug that targets the fourth enzyme of the *de novo* pathway, *Pf*DHODH (Figs 1, 7C and S7B). Control parasites not exposed to DSM1 grew equally well with or without 50 μM uracil, indicating that uracil addition alone does not affect parasite growth. As expected, DSM1 treatment inhibited parasite growth, which was fully rescued by uracil, confirming that the FUR1 bypass is functional. We found that FUR1-mediated uracil salvage could also rescue parasites treated with the mitochondrial complex III inhibitor atovaquone, which indirectly blocks pyrimidine synthesis by blocking ubiquinone regeneration required for *Pf*DHODH activity (S7C Fig). By contrast, uracil supplementation did not rescue parasites from growth inhibition by blasticidin-S or azithromycin, which target protein translation, demonstrating the specificity of the pyrimidine bypass system (S7D and S7E Fig).

In the *Pf*Mev^FUR1^ background, we successfully generated a Δ*Pf*DHO line in the presence of uracil using the same CRISPR approach that had failed in the parental *Pf*Mev line (S8 Fig). Withdrawal of uracil led to a rapid arrest in the growth of Δ*Pf*DHO parasites, demonstrating that *Pf*DHO is indeed essential for pyrimidine synthesis (Fig 7D).

### Pyrimidine biosynthetic enzymes do not have essential moonlighting functions

*Pf*ATC and *Pf*CPSII contain unusual features that raise the possibility of additional, noncanonical functions. Supporting this idea, studies in the related apicomplexan parasite *Toxoplasma gondii*, which can synthesize and salvage pyrimidines, indicate that enzymes in the pyrimidine biosynthesis pathway may serve secondary roles. Replacement of the DHODH gene in *T. gondii* with a catalytically inactive version was lethal despite intact pyrimidine salvage, pointing to a potential structural or nonenzymatic role for DHODH [56].

To test whether *Pf*ATC, *Pf*CPSII, and *Pf*DHODH serve similarly expanded roles in *P. falciparum*, we utilized the FUR1/uracil system to bypass the parasite’s reliance on its *de novo* pyrimidine synthesis pathway. The mCh-FUR1 expression plasmid was integrated into the *att*B sites in the existing *Pf*ATC^CD^ and *Pf*CPSII^CD^ parasite lines (S9A and S9B Fig). We also established an NF54^attB^ parasite line expressing mCh-FUR1 (NF54^FUR1^) (S10A and S10B Fig). Attempts to generate HA-tagged *Pf*DHODH conditional knockdown parasites in the NF54^FUR1^ background were unsuccessful. Since peptide tags can affect the structure and function of some proteins, we repeated the transfections using a TetR-DOZI plasmid modified to omit the addition of the C-terminal HA tag to *Pf*DHODH. This approach successfully yielded a *Pf*DHODH^CD^ parasite line (S10C and S10D Fig). All three conditional knockdown lines were maintained in media containing aTet.

**Fig 8:**
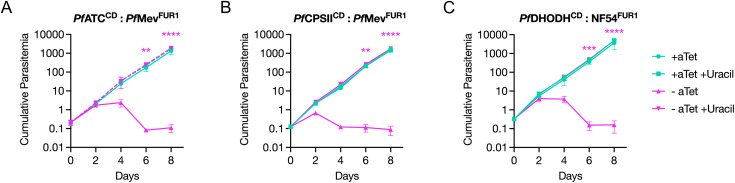
*Pf*ATC, *Pf*CPSII and *Pf*DHODH are dispensable in the presence of a pyrimidine salvage pathway. **(A)** *Pf*ATC^CD^, **(B)** *Pf*CPSII^CD^, and **(C)** *Pf*DHODH^CD^ parasites expressing mCh-FUR1 were monitored for their growth kinetics in the presence or absence of aTet and uracil by flow cytometry for 8 days. Parasite death resulting from the removal of aTet could be prevented with uracil supplementation. Growth curves were generated from two biological experiments conducted in quadruplicate. The means of technical replicates from each experiment were used for plotting and statistical analysis using GraphPad Prism 10 (GraphPad Software, Inc). Cumulative parasitemia was plotted on a log-scale Y-axis with standard deviation. Data were analyzed using two‑way ANOVA, followed by Bonferroni’s multiple‑comparison correction, with significant differences noted between -aTet and -aTet +Uracil conditions (**, P ≤ 0.01; ***, P ≤ 0.001; ****, P ≤ 0.0001).

If *Pf*ATC, *Pf*DHO, and *Pf*DHODH play essential roles beyond pyrimidine synthesis, we would expect that the FUR1/uracil salvage mechanism would not compensate for their loss. To test this, parasites from the three transgenic lines were divided into media with or without aTet and uracil. Parasite growth was monitored over an 8-day period. As expected, all three lines failed to grow in the absence of aTet and uracil (Fig 8A, 8B and 8C). However, the presence of uracil in aTet-deprived cultures completely restored the growth of the *Pf*ATC^CD^, *Pf*CPSII^CD^, and *Pf*DHODH^CD^ parasites, indicating that the essential function of these enzymes is confined to pyrimidine synthesis during asexual blood-stage replication.

Next, to assess whether these proteins participate in multienzyme assemblies analogous to mammalian CAD, we leveraged the FLAG-tagged lines in combination with the uracil bypass system to examine their native protein complexes by blue native PAGE (BN-PAGE). Immunoblotting with α-FLAG antibodies detected several distinct bands in *Pf*ATC^CD^ and *Pf*CPSII^CD^ lysates from parasites grown in the presence of aTet, but not in uracil-supplemented cultures lacking aTet (S11A Fig). A prominent band was detected in *Pf*ATC^CD^ lysates that could represent the homotrimeric complex, along with additional higher-molecular weight species (S11A and S11B Fig). *Pf*CPSII-2× FLAG migrated as large complexes, suggestive of oligomerization or association with other proteins, as well as small species (S11A Fig). However, we did not observe consistent co-migration patterns supporting the formation of a complex containing both *Pf*ATC and *Pf*CPSII. No specific banding pattern was observed for *Pf*DHO, which could be due to incomplete solubilization or limited accessibility of the C-terminal FLAG epitope in the native protein (S11C Fig). These results suggest that individual enzymes exist in larger assemblies, but there is no clear indication that they form a shared, stable complex under the conditions tested.

### *Pf*ATC is not the target of Torin 2 in asexual parasites

*Pf*ATC has been proposed as a target of Torin 2, a lead compound identified from a small-molecule screen against gametocytes that also shows potent activity against asexual parasites and purified recombinant *Pf*ATC [68,69]. To determine whether Torin 2 inhibits *Pf*ATC in asexual parasites, dose-response assays were performed using FUR1-expressing *Pf*ATC^CD^ parasites in the presence or absence of uracil. If *Pf*ATC is the relevant target, enabling pyrimidine salvage with uracil supplementation would be expected to rescue parasite growth. However, the IC_50_ values were nearly identical under both conditions (+Uracil: 0.41 nM [0.4 – 0.45 nM]; -Uracil: 0.42 nM [0.38 – 0.43 nM]), indicating no shift in drug sensitivity (S12 Fig). These findings suggest that Torin 2 does not inhibit *Pf*ATC in asexual *P. falciparum* parasites. Because the *Pf*ATC^CD^:*Pf*Mev^FUR1^ line was established in a parasite background that is defective in gametocyte production, this analysis could not be extended to gametocytes.

## Discussion

In this report, we applied molecular genetic approaches to study multiple enzymes in the pyrimidine biosynthetic pathway of *P. falciparum*. Unlike their multifunctional counterparts in mammalian cells, these enzymes are encoded as discrete gene products in *P. falciparum*, with some containing unknown features that could indicate additional functions or distinct modes of regulation [11,15,33,37].

To determine the importance of these parasite-specific differences, we initially focused our analysis on *Pf*ATC, which has been structurally resolved and characterized *in vitro* [20,57]. Using a conditional knockdown approach, we confirmed that *Pf*ATC is essential for parasite survival (Fig 4). Contrary to predictions that its N-terminal extension functions as an apicoplast-trafficking peptide, antibodies raised against *Pf*ATC showed that the protein localizes primarily to the cytoplasm (Fig 3D and 3E). Interestingly, both the N-terminal peptide alone and full-length *Pf*ATC expressed as a second copy from a dispensable locus were able to direct a fluorescent reporter to the parasite periphery, suggesting that low-level or stage-specific trafficking to this compartment may occur (Fig 3A and 3B). We have not examined if peripheral *Pf*ATC associates with the parasite plasma membrane or is present in the parasitophorous vacuole. This observed discrepancy in localization between native *Pf*ATC and the tagged transgenes could reflect variations in expression level or timing arising from the use of a heterologous promoter (Figs 3 and S5). Deletion of the N-terminus from the second copy abolished peripheral localization and diminished its ability to rescue parasites depleted of endogenous *Pf*ATC (Fig 5). This indicates that the N-terminal region contributes to *Pf*ATC function, potentially by influencing protein targeting, stability, or interactions with other proteins or membranes. If some endogenous *Pf*ATC indeed localizes to the parasite periphery, a plausible model is that its N-terminal region acts as a membrane anchor to organize *Pf*CPSII and *Pf*DHO into a higher-order complex analogous to the multifunctional architecture of human CAD, thereby enhancing metabolic efficiency [34]. Notably, a recent spatial proteomic map of *P. falciparum* schizonts assigned *Pf*ATC to the plasma membrane, lending circumstantial support to this model [70]. BN-PAGE analysis of native *Pf*ATC and *Pf*CPSII revealed the presence of high-molecular-weight complexes, but with no evidence of co-migrating species under the conditions tested (S11 Fig). It is possible that interactions between the three enzymes are transient and may require cross-linking strategies for detection.

*Pf*ATC has been the focus of several drug discovery efforts [57,68,71–73]. Structural analyses of the apo (T-state) and liganded (R-state) forms of the enzyme laid the groundwork for rational drug design aimed at disrupting the transition between the two states [57]. More recent fragment-based studies have revealed a previously unrecognized allosteric pocket in *Pf*ATC [72]. A series of compounds selectively binding to this site were found to block parasite growth in red blood cells with limited effects against human cells [72]. Together with our genetic validation of *Pf*ATC essentiality, these advances strengthen the rationale for targeting this enzyme for antimalarial drug development. Complementing these structure-guided approaches, Torin 2 was identified as a potential inhibitor of *Pf*ATC from a small-molecule screen against gametocytes [68,69,73]. While our data do not support *Pf*ATC inhibition by Torin 2 in asexual parasites (S12 Fig), we cannot preclude stage-specific targeting in gametocytes.

*Pf*CPSII, which catalyzes the first reaction in the pathway, is among the largest known members of this enzyme class due to two insertions of unknown function [15]. As predicted, *Pf*CPSII was found to be essential for parasite growth (Fig 6D). C-terminal epitope tagging revealed that *Pf*CPSII is cytoplasmic (Fig 6B) and produces multiple bands on an immunoblot, indicative of protein processing or degradation (Fig 6C). Proteolytic processing may be a necessary step in CPSII maturation, generating different enzymatic or regulatory subunits from the large precursor protein. Future work involving protein truncation analysis will be important to define the functional contributions of the insertions.

*Pf*DHO was previously predicted to be dispensable, albeit with a fitness cost, in a genome-wide transposon mutant screen [41]. One possible explanation for this phenotype is that carbamoyl aspartate undergoes slow, spontaneous hydrolysis to form dihydroorotate in the absence of *Pf*DHO, thereby permitting limited growth. To directly test the essentiality of *Pf*DHO, we bypassed the parasite’s reliance on *de novo* pyrimidine biosynthesis by introducing the yeast uracil phosphoribosyltransferase FUR1, which converts uracil to UMP (Fig 7A) [67]. This system allowed us to modulate the activity of the salvage enzyme by adjusting uracil concentrations in the culture medium. Consistent with prior work from Painter *et al*. [67], we demonstrated that uracil supplementation rescued parasites from inhibition by DSM1 or atovaquone (Figs 7C, S7B and S7C). Using this approach, we showed that *Pf*DHO is essential, as knockout parasites survived only when supplied with exogenous uracil (Fig 7D). Functionally, FUR1 achieves the same outcome as yeast DHODH (yDHODH) in circumventing ubiquinone-dependent pyrimidine synthesis, but its uracil-driven activity provides a controllable alternative to the constitutive bypass from yDHODH. This makes the FUR1 system a versatile genetic tool, both as a selectable marker and for screening compounds that target the mETC and pyrimidine biosynthetic enzymes.

We next applied this system to investigate whether the additional domains in *Pf*ATC and *Pf*CPSII confer functions beyond their canonical enzymatic roles. By introducing FUR1 into conditional knockdown lines and regulating uracil availability, we could selectively rescue pyrimidine synthesis and assess whether these enzymes contribute to parasite fitness through other mechanisms. In both cases, uracil supplementation fully restored growth, indicating that *Pf*ATC and *Pf*CPSII are essential at this stage of parasite development solely for their roles in *de novo* pyrimidine biosynthesis (Fig 8A and 8B). We extended this approach to determine if *Pf*DHODH, like its homolog in *T. gondii* [56], also possesses a non-enzymatic role. In *P. falciparum*, however, *Pf*DHODH appears to function exclusively in pyrimidine synthesis during parasite replication in red blood cells (Fig 8C).

Overall, our findings strengthen the case for prioritizing the pyrimidine biosynthesis pathway in antimalarial drug discovery. The distinct domain architecture of its constituent enzymes makes them attractive for the development of highly specific inhibitors. Particularly, it will be of interest to further study *Pf*CPSII to determine if its unusual insertions confer distinct mechanisms of regulation or maturation that could be exploited therapeutically.

## Methods

### Parasite culture

*P. falciparum* NF54^attB^ and *Pf*Mev^attB^ parasites were cultured in red blood cells (RBCs) at 2% hematocrit using complete medium supplemented with AlbuMAX II (CMA) [61,74]. CMA was prepared from RPMI-1640 medium with L-glutamine (USBiological Life Sciences, R8999) and further supplemented with 0.2% sodium bicarbonate, 12.5 μg/mL hypoxanthine, 20 mM HEPES, 5 g/L AlbuMAX II (ThermoFisher Scientific, 11021037), and 25 μg/mL gentamicin. Cultures were maintained in flasks or plates at 37°C under a gas mixture of 94% N₂, 3% O₂, and 3% CO₂.

### Plasmid construction

Various *Pf*ATC-mCherry expression vectors were generated using the previously described p15-*Ec*DPCK-mCherry plasmid as a backbone [75]. To replace the *Ec*DPCK gene, the plasmid was digested with *Avr*II and *Bsi*WI. Coding sequences for *Pf*ATC_36_, *Pf*ATC_375_, or *Pf*ATC_Δ36_ were amplified from *P. falciparum Pf*Mev^attB^ cDNA using primers Pyr_001 - Pyr_004 containing flanking *Avr*II and *Bsi*WI sites (S1 File). The PCR products were then digested with the corresponding enzymes and ligated into the linearized p15-mCherry vector.

The backbone from plasmid p15-*Ec*DPCK-mCherry [75] was also used to generate the p15-mCherry-FUR1 plasmid by replacing the region encoding *Ec*DPCK-mCherry with nucleotides encoding mCherry-FUR1. The mCherry fragment was amplified from the p15-*Pf*ATC_36_-mCherry vector using primers Pyr_005 and Pyr_006 (S1 File), containing *Avr*II sites, and the *S. cerevisiae* uracil phosphoribosyltransferase gene FUR1 was synthesized by LifeSct LLC (Rockville, MD) and amplified using primers (Pyr_007 and Pyr_008) containing *Bsr*GI and *Xho*I restriction sites (S1 File).

To knock down gene expression, we used the pKD plasmid and its derivatives, which contain the TetR-DOZI expression cassette and a 10× aptamer array [64]. Homology arms 1 (HA1) and 2 (HA2) for *Pf*ATC, *Pf*CPSII, and *Pf*DHODH were amplified using primers listed in S1 File. The reverse primers were designed to contain a recodonized sequence to eliminate homology with the guide RNA. Additional HA1 reverse primers were used for *Pf*ATC and *Pf*CPSII to restore the native coding sequence downstream of the recodonized region, but not including the stop codon. HA2 amplicons were cloned into the *Asc*I and *Eco*RV sites in pKD, and the final HA1 amplicons were ligated into *Eco*RV and *Aat*II sites. For *Pf*DHO, a synthetic fragment consisting of HA2-recoded HA1–3× FLAG flanked by *Asc*I and *Psp*OM1 sites (LifeSct LLC) was cloned into the pTDN plasmid, a derivative of pKD (S1 and S2 Files). pTDN has three changes relative to pKD. Unnecessary attP sites were removed by digestion with *Eco*RI, followed by religation of the plasmid. The TetR-DOZI coding sequence was replaced with a synthesized sequence (LifeSct LLC, S2 File) to remove unwanted endonuclease sites and inserted into the *Avr*II and *Nhe*I sites in pKD. Finally, the blasticidin deaminase gene was replaced with a synthetic neomycin resistance gene (LifeSct LLC, S2 File) via digestion with *Age*I and *Ngo*MIV, followed by ligation. To create a version of the *Pf*DHODH-pKD plasmid lacking the C-terminal 2× FLAG tag, the HA1 sequence along with the stop codon was amplified using primers encoding *Eco*RV and *Psp*OM1 sites (S1 File), digested with the corresponding enzymes, and cloned into the same sites in the *Pf*DHODH-pKD vector, effectively removing the tag.

The gene deletion construct for *Pf*DHO was generated using the pRSng-BD repair plasmid [76]. The HA1 and HA2 regions were amplified using primers listed in S1 File. The HA1 sequence was inserted into the *Not*I site and HA2 into the *Ngo*MIV site of the plasmid.

To construct plasmids encoding guide RNAs for gene knockdown or knockout experiments, the pCasG-BD-LacZ vector (where “BD” denotes blasticidin deaminase, which confers resistance to blasticidin-S) was first digested with the restriction enzyme *Bsa*I [64]. Custom guide RNA sequences were synthesized as complementary oligonucleotides (S1 File), annealed to form duplexes, and subsequently cloned into the digested vector by ligation-independent cloning with In-Fusion (Takara, 639650).

All restriction enzymes and T4 DNA Ligase (M0202S) were sourced from New England Biolabs.

### Parasite transfections

To generate transgenic *P. falciparum* lines expressing *Pf*ATC-mCherry variants and mCherry-FUR1, 350 μL volumes of RBCs were electroporated using the GenePulser XCell system (Bio-Rad) with: 50 μg of the pINT plasmid [62] that expresses the Bxb1 integrase, and 65 μg of the p15 plasmid with an expression cassette for the mCherry fusion gene and the dihydrofolate reductase (DHFR) resistance marker that confers resistance to the compound WR99210. Electroporated RBCs were mixed with 1 mL of parasite culture at 2–3% parasitemia and maintained in 10 mL of CMA for 2 days, then transferred to CMA containing 2.5 nM WR99210 (Jacobus Pharmaceutical Company, Inc.) for 7 days. Cultures were then returned to drug-free CMA until parasites became detectable by blood smear, at which point WR99210 was reintroduced.

For knockdown lines, RBCs were electroporated with 65 μg each of the pKD/pTDN plasmid and the corresponding pCasG-BD-gRNA plasmid. Following electroporation, the RBCs were treated as above and maintained in CMA with 0.5 μM anhydrotetracycline (aTet; Cayman Chemical: 10009542). Cultures were placed under selection with either 2.5 μg/mL blasticidin-S (for pKD; Corning: 30–100-RB) or 0.5 mg/mL G418 (for pTDN; Corning: 61–234-RG) along with aTet for 7 days. They were then maintained in CMA and aTet until parasites reemerged, after which drug selection was resumed.

For *Pf*DHO gene deletion, RBCs were electroporated with the pRSngBD-*Pf*DHO plasmid and the corresponding pCasG-gRNA plasmid, then mixed with 1 mL of *Pf*Mev^FUR1^ parasites at 2–3% parasitemia in CMA supplemented with 50 μM uracil. Two days post transfection, the culture was selected with 2.5 μg/mL blasticidin-S in the presence of uracil for 7 days, followed by maintenance in CMA and uracil until parasite reappearance, at which point blasticidin-S was added back to the medium.

Clonal parasites for all transgenic lines were obtained by limiting dilution in 96-well plates. The genotypes of transgenic parasites were confirmed by PCR using primers listed in S1 File.

### Growth assays

To assess the growth kinetics of various parasite lines under different treatments, the parasites were washed three times to remove existing drugs and supplements from the media, diluted to 0.5% parasitemia in 2% hematocrit, and distributed into the appropriate selective media conditions. Cultures were seeded in 200 μL volumes per well in 96‐well plates (ThermoFisher Scientific, 267427), and the parasitemia was determined by flow cytometry every other day over an 8-day period, unless stated otherwise. Immediately after each sampling point, cultures were diluted eight-fold to prevent overgrowth. For the data represented in Figs 6D and S6E, cultures were instead diluted four-fold and ten-fold every two days, respectively. All source data for the growth curves are available in S3 File.

To prepare samples for flow cytometry, 20 μL volumes of parasite cultures were taken from each well, diluted 1:5 in PBS and stored at 4 °C in 96-well plates until analysis. Parasites were stained by adding 10 μL of the 1:5 dilutions to a 96-well plate containing 100 μL of 1× SYBR Green (Invitrogen, S7563) in PBS, followed by a 30-minute incubation in the dark on a plate shaker. Afterwards, 150 μL of PBS was added to reduce background fluorescence from unbound dye. Stained cells were analyzed using an Attune NxT Flow Cytometer (ThermoFisher Scientific), operated at a flow rate of 50 μL/minute to collect 10,000 events per sample. Each condition was tested in quadruplicate.

### *P. falciparum* growth inhibition assays

Inhibitory activities of DSM1 (15 mM stock solution in DMSO), blasticidin-S (5.45 mM stock solution in water) and azithromycin (Sigma-Aldrich: 75199; 10 mM stock solution in DMSO) were tested against NF54^FUR1^ parasites in the presence or absence of 50 μM uracil. Torin 2 (Cayman Chemical: 14185; 5 mM stock solution in DMSO) dose-response experiments were similarly conducted using *Pf*ATC^CD^ parasites expressing FUR1 in medium with or without uracil.

For all assays, parasites were diluted to 1% parasitemia at 1.5% hematocrit and exposed to serial drug dilutions (DSM1: 0–15 μM; blasticidin-S: 0–163.5 μM; azithromycin: 0–10 μM; Torin 2: 0–102.4 nM). For compounds prepared in DMSO, the final DMSO concentration did not exceed 0.1%. After 72 hours of incubation under standard culture conditions (extended to 96 hours for azithromycin to account for the apicoplast delayed-death phenotype [77]), parasitemia was quantified by flow cytometry using the Attune NxT Flow Cytometer as described above, or the Guava easyCyte Flow Cytometer (Cytek) operated at a medium flow rate to collect 10,000 events. All source data are available in S3 File.

### Antibody generation and validation

DNA encoding amino acid residues 38–375 of *Pf*ATC was PCR-amplified from *P. falciparum* NF54^attB^ genomic DNA using primers Pyr_064 and Pyr_065 (S1 File). The product was digested with *Eco*RI and *Hin*dIII and cloned into the same sites of the pMALcHT *Escherichia coli* expression vector [78] using In-Fusion ligation-independent cloning to yield the pMALcHT-*Pf*ATC expression plasmid. This plasmid was cotransformed with the pRIL plasmid purified from BL21-CodonPlus (DE3)-RIL competent cells (Agilent Technologies), and plasmid pKM586 encoding TEV protease [79]. When coexpressed, the TEV protease allows for *in vivo* cleavage of the N-terminal maltose binding protein (MPB) from the MBP-6×His-*Pf*ATC.

*E. coli* cells transformed with the plasmids above were grown in LB medium at 37 °C to an OD_600_ of 0.6, at which point protein expression was induced with 0.4 mM isopropyl β-thiogalactopyranoside (IPTG). Cells were harvested after 8 hours of growth at 16 °C. Cell pellets were resuspended in 20 mL of lysis buffer (10 mM Tris-HCl, pH 8.0, 100 mM NaCl, 2.5 µg/mL DNase I, and 1 mg/mL lysozyme) per liter of cell culture. The resuspended cells were lysed by sonication, and the lysate was clarified by centrifugation at 4 °C for 20 minutes at 20,000 × g. The supernatant was loaded onto a Ni^2+^-charged 5 mL HiTrap chelating HP column (Cytiva) pre-washed with equilibration buffer (10 mM Tris-HCl, pH 8.0, 100 mM NaCl). The column was washed with 2 column volumes of equilibration buffer before connecting to a 5 mL MBPTrap HP column (Cytiva) pre-washed with equilibration buffer (the MBPTrap column will remove any uncut fusion protein). The bound proteins from the HiTrap column were eluted using a 0–400 mM imidazole gradient in equilibration buffer, and further purified by size-exclusion chromatography using a HiPrep 26/60 Sephacryl S-100 HR size-exclusion column (Cytiva) equilibrated with phosphate-buffered saline (PBS, pH 7.4).

Pure recombinant *Pf*ATC was used to generate rat antiserum (Cocalico Biologicals Inc). Two rats were immunized with *Pf*ATC in Complete Freund’s Adjuvant and were boosted at weeks 2, 3, and 7. Terminal bleeds were collected on day 56. Anti-*Pf*ATC IgG was purified from antiserum using a *Pf*ATC affinity column as described previously [80]. A total of 10 mg of α-*Pf*ATC IgG was concentrated to 0.25 mg/mL and stored at −80 °C in storage buffer (PBS, 40% glycerol, 0.02% NaN₃).

To validate the α-*Pf*ATC antibody, we utilized the *Pf*ATC^CD^:*Pf*Mev^FUR1^ parasites, taking advantage of the fact that these parasites remain viable with uracil supplementation even after *Pf*ATC knockdown. Western blot analysis with the α-*Pf*ATC antibody revealed loss of a ~ 45 kDa band upon aTet removal, corresponding to endogenous *Pf*ATC-2× FLAG (S13A Fig). Notably, stripping and reprobing the same blot with an α-FLAG antibody confirmed that this band is present at the same molecular weight in +aTet lysates and absent in −aTet lysates. Similarly, immunofluorescence microscopy using the α-*Pf*ATC antibody detected a robust signal in parasites maintained with aTet, which was largely lost following aTet withdrawal, confirming antibody specificity (S13B and S13C Fig).

### SDS-PAGE, BN-PAGE and Western Blotting

Parasite pellets were collected by centrifugation at 500 × g for 5 minutes at room temperature (RT). To release parasites from RBCs, the pellets were treated with 0.15% saponin for 5 minutes at RT, followed by three washes in 1× PBS. The resulting parasite pellets were solubilized in NuPAGE LDS sample buffer (Invitrogen: NP0007) supplemented with 10% β-mercaptoethanol and heated at 95 °C for 5 minutes. Protein extracts were separated on 4–12% Bis-Tris SDS-PAGE gels (Invitrogen: NP0322) and transferred onto nitrocellulose membranes.

For BN-PAGE, parasite pellets were prepared using a PAGE sample kit (Invitrogen: BN2008). Briefly, parasite pellets were solubilized at 12.5 mg/25 µL in NativePAGE sample buffer with 2.5% digitonin and incubated on ice for 15 minutes. Solubilized pellets were centrifuged at 16,000 x g for 15 minutes at 4 °C. The supernatants were recovered, supplemented with 1 µL 5% Coomassie-blue loading buffer and separated on a NativePAGE 3–12% protein gel (Invitrogen: BN1001BOX) for approximately 2 hours at 150 V. Proteins were transferred to PVDF membranes. Bovine heart mitochondria (BHM; Abcam: AB1103382MG) and NativeMark Unstained Protein Standard (Invitrogen: LC0725) were used as native protein ladders. Differences in migration between BHM and the NativeMark standard on BN-PAGE likely reflect differences in protein composition and Coomassie dye binding under native conditions [81]; both were included for complementary reference.

Membranes were blocked with 5% non-fat milk or bovine serum albumin (BSA) in 1× PBST (PBS with 0.1% Tween 20) and incubated overnight at 4 °C with the appropriate primary antibodies at the following dilutions in milk/PBST: 1:20,000 mouse α-FLAG M2 monoclonal antibody (Millipore Sigma: F3165) 1:2,500 rat α-HA monoclonal antibody (3F10, Roche: 11867423001), 1:250 affinity purified rat α-*Pf*ATC polyclonal antibodies (this study), 1:500 rat α-*Pf*ATC antisera, or 1:1,000 rabbit α-*Pf*BiP antisera (BEI Resources: MRA-1246) [82,83]. After washing, blots were incubated at room temperature for 1 hour with HRP-conjugated secondary antibodies from GE Healthcare (1:10,000 anti-mouse, 1:5,000 anti-rat, or 1:5,000 anti-rabbit). Signal was developed using SuperSignal West Pico Chemiluminescent Substrate (ThermoFisher Scientific: 34580) and visualized on X-ray film or a ChemiDoc imaging system (Bio-Rad). To assess loading controls, blots were stripped using 200 mM glycine (pH 2.0) for 5 minutes, then reprobed overnight at 4 °C.

### Microscopy

For live fluorescence microscopy, 100 μL of parasite culture was stained with 1 μg/mL DAPI (ThermoFisher Scientific: 62248) for 30 minutes at 37 °C, washed three times with CMA, and resuspended in 10 μL CMA for mounting on glass slides. Coverslips were sealed with wax, and images spanning 5 μm (0.2 μm steps) were acquired using a Zeiss AxioImager M2 microscope. For immunofluorescence assays, parasites were fixed in 4% formaldehyde and 0.0075% glutaraldehyde in PBS for 30 minutes, permeabilized with 1% Triton X-100, and blocked in 3% BSA for 2 hours. Cells were incubated overnight at 4 °C with 1:200 affinity purified rat α-*Pf*ATC antibodies (this study), 1:500 rabbit α-ACP antibodies [84], 1:500 rabbit α-BiP antibody (BEI Resources: MRA-1246), or 1:500 mouse α-FLAG M2 antibody (Millipore Sigma: F3165), followed by PBS washes and incubation with Alexa Fluor-conjugated secondary antibodies: 1:300 goat anti-rat 488 (ThermoFisher Scientific: A-11006), 1:500 goat anti-rabbit 594 (ThermoFisher Scientific: A-11037), or 1:500 goat anti-mouse 488 (ThermoFisher Scientific: A-11001). Samples were mounted in ProLong Gold Antifade reagent with DAPI (Invitrogen: P36935) and sealed with nail polish. For both live and fixed imaging, Z-stacks were deconvolved using VOLOCITY software (PerkinElmer), and representative single-plane images were reported.

## Supporting information

S1 Fig Generation of transgenic P*. falciparum* lines with mCherry-tagged proteins of interest. **(A)** The schematic depicts integration of the p15.mCherry plasmid into the parasite genome. The p15.mCherry plasmid was inserted into the P230p or CG6 locus of *P. falciparum Pf*Mev^attB^ or NF54^attB^ parasites, respectively, by Bxb1 integrase-mediated recombination at the plasmid *att*P and genome *att*B sites. The modified locus contains the entire plasmid flanked by new *att*L and *att*R sites that are generated by recombination. Arrows indicate the positions of primers used for diagnostic PCR. The Cam/HOP bidirectional promoter drives expression of the gene of interest (GOI) fused to mCherry and the selection marker hDHFR (human dihydrofolate reductase). **(B)** Primer pairs for verifying integration of the p15.mCherry plasmid into the P230p genomic locus are listed along with the expected sizes of the PCR products. Integration of **(C)** p15.*Pf*ATC_36_-mCherry, and **(D)** p15.*Pf*ATC_375_-mCherry plasmids in *Pf*Mev^attB^ (Parent, ‘P’) parasites was confirmed by PCR amplification (attL and attR products). The intact P230p locus (attB product) was detected only in the parent (blue) and not in the transgenic lines (orange).
(TIF)

S2 Fig Generation and validation of *Pf*ATC antisera. **(A)** *Pf*ATC (amino acids 38–375) was fused to an N-terminal 6× His tag for recombinant protein expression and purification. **(B)** Purified recombinant *Pf*ATC resolved on SDS-PAGE under reducing conditions and stained with Coomassie blue. **(C)** Purified *Pf*ATC was probed with pre-bleed (day 0), test bleed (day 35), or final bleed (day 56) antisera (1:500) from a rat immunized with recombinant *Pf*ATC. The black arrowhead indicates the specific band for *Pf*ATC, while the orange arrowhead marks a non-specific band detected even with pre-bleed antisera.
(TIF)

S3 Fig Schematic of knockdown plasmid insertion into the 3’ UTR of target genes. **(A)** The pKD plasmid was linearized by digestion with *Eco*RV prior to transfection. Homologous regions between the linearized vector and the Parent (P) locus are indicated by dotted lines. The Hsp86 5’ UTR (untranslated region) contains a promoter element that drives TetR-DOZI expression. Arrows indicate the positions of primers used for diagnostic PCR. ‘TAG’ refers to the 2× FLAG epitope tag appended to the target protein, and 10×Apt refers to the aptamer array inserted in the 3’ UTR of the target gene. HA1 and HA2 are the homology arms utilized for recombination. This schematic also applies to the pTDN plasmid. **(B)** Primer pairs for verifying integration of the pKD-*Pf*ATC plasmid into the 3’ UTR of the *Pf*ATC gene are listed, along with the expected sizes of the PCR products. **(C)** Integration of the pKD-*Pf*ATC plasmid was confirmed by PCR amplification of the 5’ and 3’ loci at the insertion site in *Pf*ATC^CD^ clonal parasites (orange). The *Pf*Mev^attB^ parent line served as a control (blue).
(TIFF)

S4 Fig Bxb1-mediated integration of *Pf*ATC-mCherry expression constructs into the *Pf*ATC^CD^ parasite genome. Genomic integration of **(A)** p15.*Pf*ATC_Δ36_-mCherry and **(B)** p15.*Pf*ATC_375_-mCherry plasmids in *Pf*ATC^CD^ (Parent, ‘P’) parasites was confirmed by PCR amplification (*att*L and *att*R products). The intact P230p locus (*att*B product) was detected only in the parent (blue) and not in the transgenic lines (orange). Refer to the schematic in S1A Fig for a detailed illustration of the integration of p15.mCherry plasmids into *att*B sites in the *P. falciparum* genome. Primer pairs for verifying integration of the p15.mCherry plasmid into the P230p locus are listed in S1B Fig along with the expected sizes of the PCR products.
(TIF)

S5 Fig Localization of endogenous and second-copy *Pf*ATC. Representative IFA images of *Pf*ATC^CD^:*Pf*ATC_375_-mCh parasites probed with α-FLAG (green) and α-mCherry (magenta) antibodies for detection of endogenous *Pf*ATC-2× FLAG and second-copy *Pf*ATC-mCherry, respectively. DAPI (blue) stains the parasite nucleus. Images represent fields that are 10 μm long by 10 μm wide.
(TIF)

S6 Fig Genotyping and characterization of *Pf*CPSII and *Pf*DHO conditional knockdown lines. **(A)** Primer pairs for verifying integration of the pKD-*Pf*CPSII plasmid and pTDN-*Pf*DHO plasmid into the 3’ UTR of *Pf*CPSII and *Pf*DHO genes, respectively, are listed along with the expected sizes of the PCR products. **(B)** Integration of the pKD-*Pf*CPSII plasmid, and **(C)** the pTDN-*Pf*DHO plasmid to create *Pf*CPSII^CD^ and *Pf*DHO^CD^ parasite lines, respectively, was validated by PCR amplification of the 5’ and 3’ loci at the genomic insertion sites (orange). The *Pf*Mev^attB^ parent (P) line served as a control (blue). Refer to S3A Fig for a schematic of knockdown plasmid insertion into the 3’ UTR of target genes. **(D)** Western blot analysis of *Pf*DHO^CD^ parasites at Day 0, 4 and 8 of aTet withdrawal, using α-FLAG antibody, showed no measurable knockdown of FLAG-tagged *Pf*DHO (black arrowhead). The α-HA antibody detecting HA-tagged api-SFG (orange arrowhead) was used as a loading control. **(E)** Growth of *Pf*DHO^CD^ parasites in the presence or the absence of aTet was monitored by flow cytometry for 8 days. Removal of aTet led to a reduction in parasite growth. The means of technical replicates from each experiment were used for plotting and statistical analysis using GraphPad Prism 10 (GraphPad Software, Inc). Cumulative parasitemia was plotted on a log-scale Y-axis with standard deviation. Data were analyzed using two‑way ANOVA, followed by Bonferroni’s multiple‑comparison correction (**, P ≤ 0.01; ***, P ≤ 0.001; ****, P ≤ 0.0001).
(TIF)

S7 Fig Parasites expressing yeast FUR1 are rescued from DSM-1 and atovaquone treatment by uracil supplementation. **(A)** Genomic integration of p15.mCherry-FUR1 in *Pf*Mev^attB^ (Parent, ‘P’) parasites was confirmed by PCR amplification (*att*L and *att*R products). The intact P230p locus (*att*B product) was detected only in the parent (blue) and not in the transgenic line (orange). Refer to S1A Fig for a detailed illustration of the integration of p15.mCherry plasmids into *att*B sites in the *P. falciparum* genome. Primer pairs for verifying integration of the p15.Cherry plasmid into the P230p locus are listed in S1B Fig along with the expected sizes of the PCR products. **(B)** *Pf*Mev^FUR1^ parasites ± uracil were exposed to a range of DSM1 concentrations for 72 h, after which parasitemia was quantified by flow cytometry. Data represent the means of three independent biological experiments with quadruplicate samples; error bars indicate standard deviations. Calculated IC₅₀ values with 95% confidence intervals are shown below the graph. **(C)** *Pf*Mev^FUR1^ parasites were treated with either 0 or 500 nM atovaquone (ATQ) in combination with increasing concentrations of uracil. Parasitemia was quantified daily by flow cytometry, with 1:10 dilutions performed every two days to prevent overgrowth. The means of technical replicates from each experiment were used for plotting and statistical analysis using GraphPad Prism 10 (GraphPad Software, Inc). Cumulative parasitemia was plotted on a log-scale Y-axis with standard deviation. Data were analyzed using two‑way ANOVA, followed by Bonferroni’s multiple‑comparison correction; ****, P ≤ 0.0001. P-value only shown for multiple comparison analysis between samples from ATQ + 50 μΜ Uracil and No ATQ + 50 μM control conditions. The addition of 50 μM uracil largely restored growth to parasites treated with ATQ. **(D-E)** *Pf*Mev^FUR1^ parasites cultured with or without uracil were exposed to various concentrations of blasticidin-S **(D)** or azithromycin **(E)** for 72 or 96 h, respectively. This was followed by measurement of parasitemia via flow cytometry. Data reflect the means of three independent biological experiments with quadruplicate samples; error bars indicate standard deviations. IC₅₀ values with 95% confidence intervals are shown below each graph.
(TIF)

S8 Fig Schematic of CRISPR/Cas9-mediated disruption of *Pf*DHO. **(A)** The pRSng plasmid encodes a blasticidin deaminase (BSD) expression cassette flanked by sequences with homology to *Pf*DHO, indicated by dotted lines. The Cam/HOP promoter drives expression of BSD. Arrows indicate the positions of primers used for diagnostic PCR. HA1 and HA2 are the homology arms utilized for recombination of pRSng into the *Pf*DHO locus in *Pf*Mev^FUR1^ parasites (Parent, ‘P’). **(B)** Primer pairs for verifying gene knockouts are listed along with the expected sizes of the PCR products. **(C)** The disruption of *Pf*DHO was confirmed by PCR amplification (Δ5’ and Δ3’ products). The intact *Pf*DHO locus (5’ and 3’ products) was detected only in the parent (blue) and not in the Δ*Pf*DHO line (orange).
(TIF)

S9 Fig Bxb1-mediated integration of mCherry-FUR1 expression constructs into the *P. falciparum* genome. Genomic integration of the p15.mCherry-FUR1 plasmid at the P230p locus in **(A)** *Pf*ATC^CD^ (Parent, ‘P’) or **(B)** *Pf*CPSII^CD^ (Parent, ‘P’) parasites was confirmed by PCR amplification of *att*L and *att*R products. The intact P230p locus (*att*B product) was detected only in the parent (blue) and not in the transgenic lines (orange). For a detailed schematic of p15.mCherry plasmid integration into *att*B sites, refer to S1A Fig. Primer pairs for verifying integration into the P230p locus are listed in S1B Fig along with the expected PCR product sizes.
(TIF)

S10 Fig Generation of a *Pf*DHODH conditional knockdown line in NF54^attB^ parasites expressing FUR1. **(A)** Primer pairs for verifying p15.Cherry-FUR1 integration into the CG6 genomic locus of NF54^attB^ parasites (Parent, ‘P’) are provided. **(B)** Genomic integration at the CG6 locus was confirmed by PCR (*att*L and *att*R products), with the intact CG6 locus (*att*B product) detected only in the parent line (blue) and not in the transgenic line (orange). Refer to S1A Fig for a detailed schematic of p15.mCherry plasmid integration into *att*B sites. **(C)** Primer pairs for verifying integration of the pKD^tagless^-*Pf*DHODH plasmid into the 3’ UTR of *Pf*DHODH are listed. **(D)** The genotype of the PDHODH^CD^ parasite line was confirmed by PCR amplification of the 5’ and 3’ loci at the genomic insertion site (orange). The NF54^FUR1^ parent (P) line served as a control (blue). Refer to S3A Fig for a schematic of knockdown plasmid insertion into the 3’ UTR of target genes.
(TIF)

S11 Fig *Pf*ATC and *Pf*CPSII form higher-order complexes. **(A)** α-FLAG immunoblot (top) and the corresponding BN-PAGE gel (bottom) of *Pf*ATC^CD^ and *Pf*CPSII^CD^ lysates from parasites cultured ± aTet. Several bands seen in +aTet conditions were not detected in - aTet samples. **(B)** Immunoblot of *Pf*ATC^CD^ + aTet sample separated by BN-PAGE, probed with α- *Pf*ATC (top) and then stripped and reprobed with α-FLAG (bottom) antibodies, revealed a prominent band along with several fainter higher molecular weight species. **(C)** α-FLAG immunoblot (top) of *Pf*DHO^CD^ and *Pf*Mev^FUR1^ (parent) lysates did not detect any specific band corresponding to *Pf*DHO-3× FLAG. The BN-PAGE gel (bottom) demonstrates comparable protein loading. Bovine heart mitochondria (BHM) and the NativeMark Unstained Protein Standard (NM) served as native molecular mass references in panels **(A)** and **(B)**, whereas panel **(C)** included only NM. The two ladders migrate differently on BN-PAGE, likely due to differences in protein composition and Coomassie dye binding under native conditions. *Note:* Apparent lane shifts between immunoblots and the BN-PAGE gel are due to minor gel misalignment during transfer.
(TIF)

S12 Fig Torin 2 likely does not inhibit *Pf*ATC in asexual *P. falciparum* parasites. *Pf*ATC^CD^ parasites expressing FUR1 were exposed to a range of Torin 2 concentrations with or without 50 μM uracil for 72 h, after which parasitemia was quantified by flow cytometry. Data represent the means of three independent biological experiments with quadruplicate samples; error bars indicate standard deviations. Calculated IC₅₀ values with 95% confidence intervals are shown below the graph.
(TIFF)

S13 Fig Validation of α-*Pf*ATC antibody specificity. **(A)** Lysates from *Pf*Mev^attB^ (control) and *Pf*ATC^CD^ parasites were probed with α-*Pf*ATC. The antibody detected a ~ 40 kDa band in *Pf*Mev^attB^ lysates (green arrow), consistent with the predicted molecular weight of *Pf*ATC (43.3 kDa). In *Pf*ATC^CD^ parasites, a slightly higher band was observed (black arrow, +aTet condition) but absent when *Pf*ATC was depleted by removal of aTet from culture media (-aTet). This higher product is consistent with the predicted 45 kDa size of the endogenously C-terminally 2× FLAG tagged *Pf*ATC. The orange arrow marks a non-specific band. The blot was stripped and reprobed with an α-FLAG antibody, which detected the same band in the + aTet *Pf*ATC^CD^ sample that was lost upon aTet withdrawal. Ponceau S staining is shown to indicate relative protein loading. **(B)** *Pf*ATC^CD^ parasites cultured under +aTet and -aTet conditions were fixed and probed with affinity-purified α-*Pf*ATC in an immunofluorescence assay. Representative images show specific staining by α-*Pf*ATC antibodies (green) in parasites grown under +aTet conditions, while no signal was detected in parasites grown under -aTet conditions. Images represent fields that are 10 μm long by 10 μm wide. **(C)** Fraction of *Pf*ATC^CD^ parasites exhibiting α-*Pf*ATC staining under +aTet and -aTet conditions. All parasites in the +aTet condition were positive for α-*Pf*ATC staining (total cells imaged = 16), while the signal was markedly reduced in the -aTet condition (total cells imaged = 36).
(TIF)

S1 File List of primers and oligonucleotides used in the study. (XLSX)

S2 File Generation of homology arms (HA) for *Pf*DHO.pTDN plasmid. (PDF)

S3 File Source data for all graphs in the study. (XLSX)
