# Supplementary figures and images for: Genetic analysis of pyrimidine biosynthetic enzymes in Plasmodium falciparum
Source: PLoS Pathog. 2026 May 27;22(5):e1014269. doi: 10.1371/journal.ppat.1014269 (PMC13232951; doi:10.1371/journal.ppat.1014269)

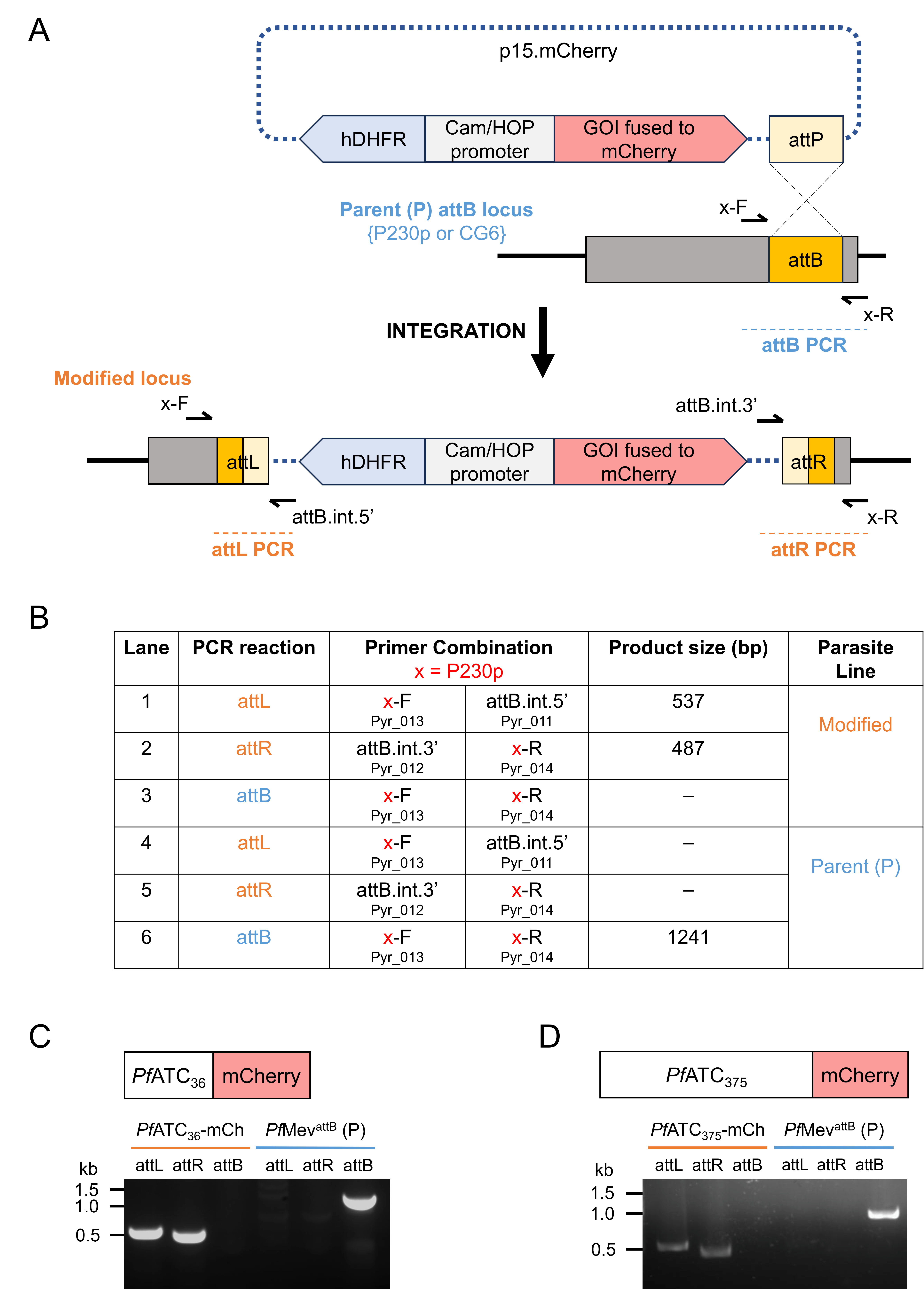

Supplement: S1 Fig — (A) The schematic depicts integration of the p15.mCherry plasmid into the parasite genome. The p15.mCherry plasmid was inserted into the P230p or CG6 locus of P. falciparum PfMevattB or NF54attB parasites, respectively, by Bxb1 integrase-mediated recombination at the plasmid attP and genome attB sites. The modified locus contains the entire plasmid flanked by new attL and attR sites that are generated by recombination. Arrows indicate the positions of primers used for diagnostic PCR. The Cam/HOP bidirectional promoter drives expression of the gene of interest (GOI) fused to mCherry and the selection marker hDHFR (human dihydrofolate reductase). (B) Primer pairs for verifying integration of the p15.mCherry plasmid into the P230p genomic locus are listed along with the expected sizes of the PCR products. Integration of (C) p15.PfATC36-mCherry, and (D) p15.PfATC375-mCherry plasmids in PfMevattB (Parent, ‘P’) parasites was confirmed by PCR amplification (attL and attR products). The intact P230p locus (attB product) was detected only in the parent (blue) and not in the transgenic lines (orange). (TIF) [file ppat.1014269.s001.tif]

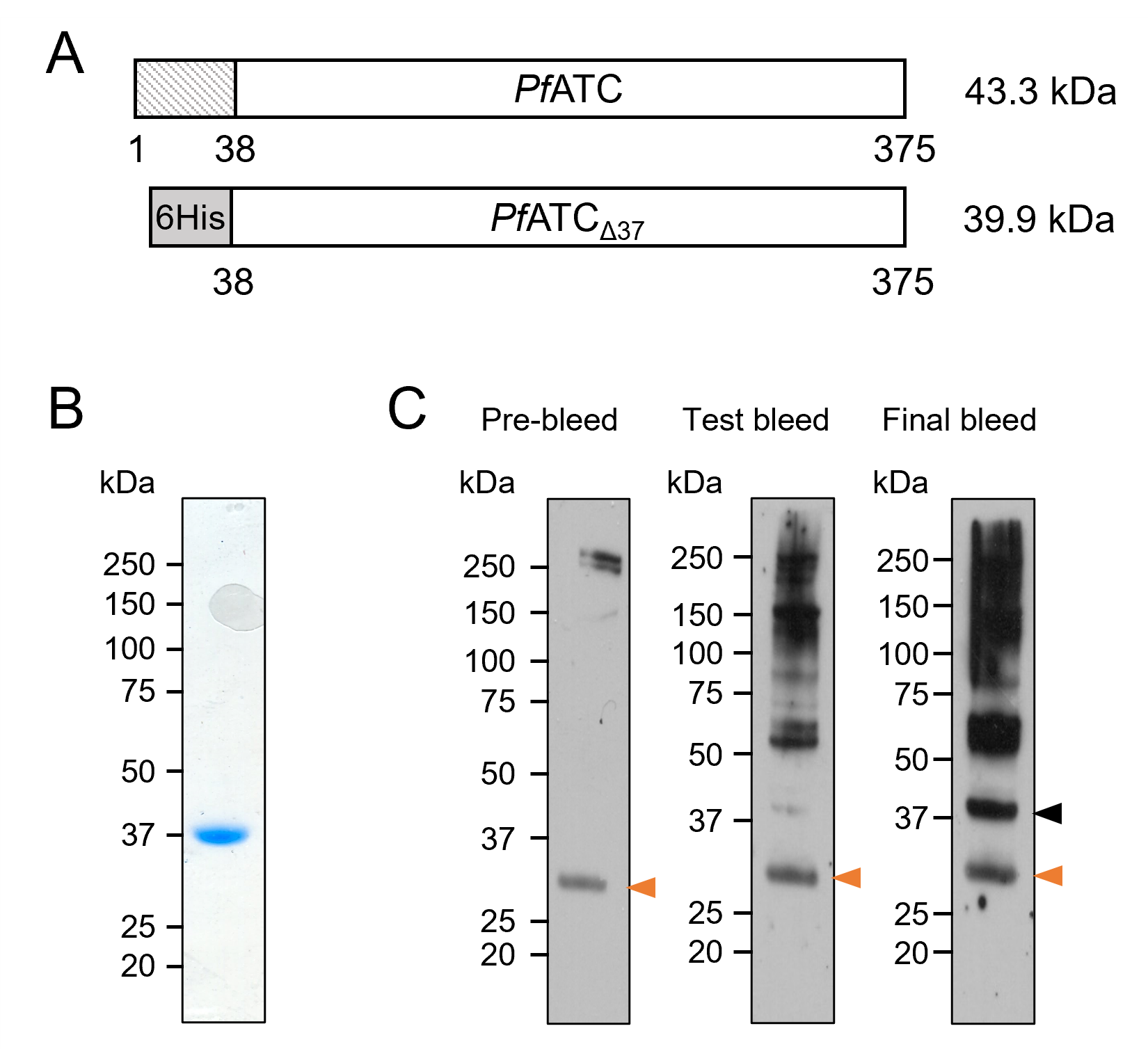

Supplement: S2 Fig — (A) PfATC (amino acids 38–375) was fused to an N-terminal 6× His tag for recombinant protein expression and purification. (B) Purified recombinant PfATC resolved on SDS-PAGE under reducing conditions and stained with Coomassie blue. (C) Purified PfATC was probed with pre-bleed (day 0), test bleed (day 35), or final bleed (day 56) antisera (1:500) from a rat immunized with recombinant PfATC. The black arrowhead indicates the specific band for PfATC, while the orange arrowhead marks a non-specific band detected even with pre-bleed antisera. (TIF) [file ppat.1014269.s002.tif]

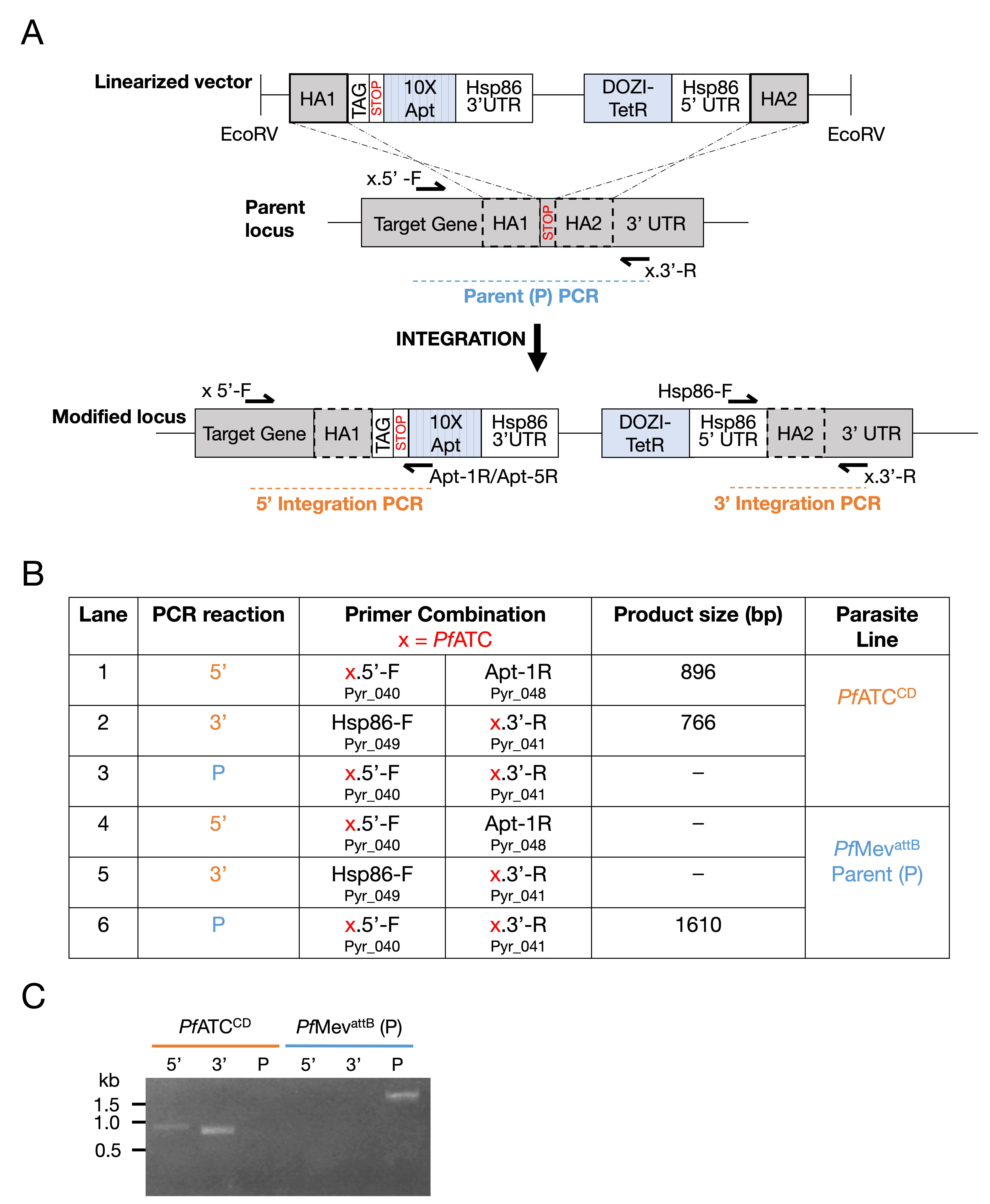

Supplement: S3 Fig — (A) The pKD plasmid was linearized by digestion with EcoRV prior to transfection. Homologous regions between the linearized vector and the Parent (P) locus are indicated by dotted lines. The Hsp86 5’ UTR (untranslated region) contains a promoter element that drives TetR-DOZI expression. Arrows indicate the positions of primers used for diagnostic PCR. ‘TAG’ refers to the 2× FLAG epitope tag appended to the target protein, and 10×Apt refers to the aptamer array inserted in the 3’ UTR of the target gene. HA1 and HA2 are the homology arms utilized for recombination. This schematic also applies to the pTDN plasmid. (B) Primer pairs for verifying integration of the pKD-PfATC plasmid into the 3’ UTR of the PfATC gene are listed, along with the expected sizes of the PCR products. (C) Integration of the pKD-PfATC plasmid was confirmed by PCR amplification of the 5’ and 3’ loci at the insertion site in PfATCCD clonal parasites (orange). The PfMevattB parent line served as a control (blue). (TIFF) [file ppat.1014269.s003.tiff]

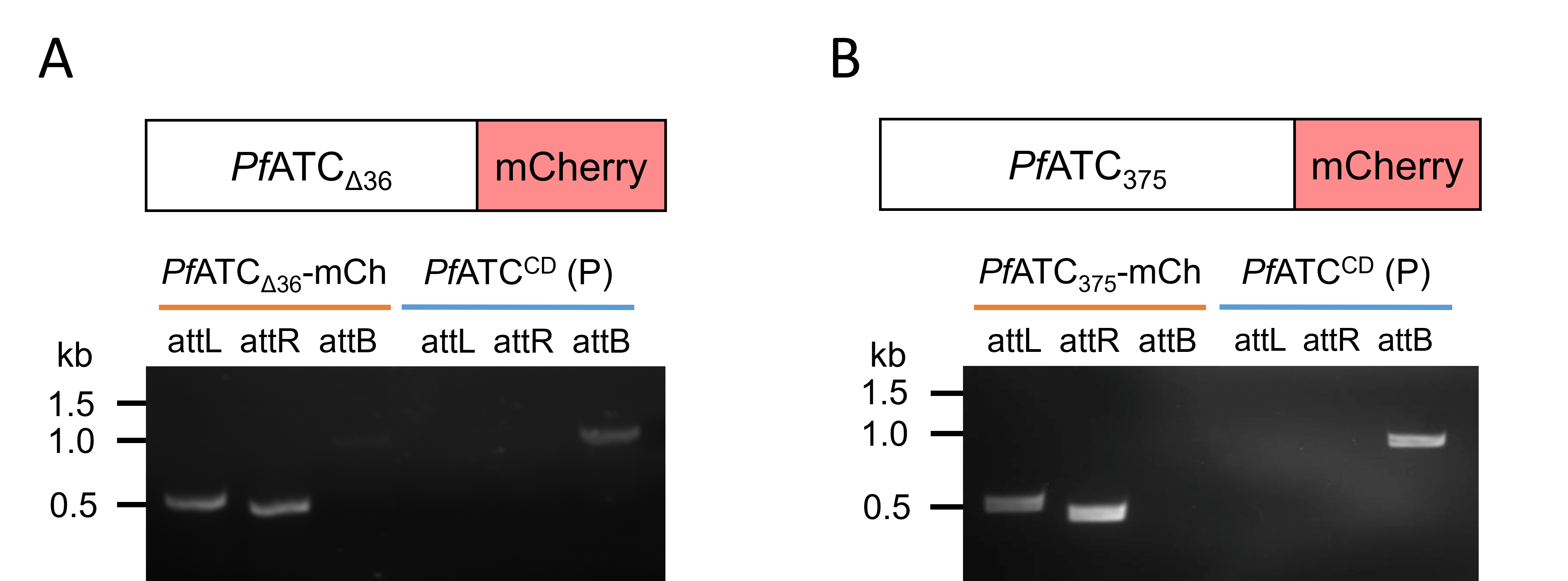

Supplement: S4 Fig — Genomic integration of (A) p15.PfATCΔ36-mCherry and (B) p15.PfATC375-mCherry plasmids in PfATCCD (Parent, ‘P’) parasites was confirmed by PCR amplification (attL and attR products). The intact P230p locus (attB product) was detected only in the parent (blue) and not in the transgenic lines (orange). Refer to the schematic in S1A Fig for a detailed illustration of the integration of p15.mCherry plasmids into attB sites in the P. falciparum genome. Primer pairs for verifying integration of the p15.mCherry plasmid into the P230p locus are listed in S1B Fig along with the expected sizes of the PCR products. (TIF) [file ppat.1014269.s004.tif]

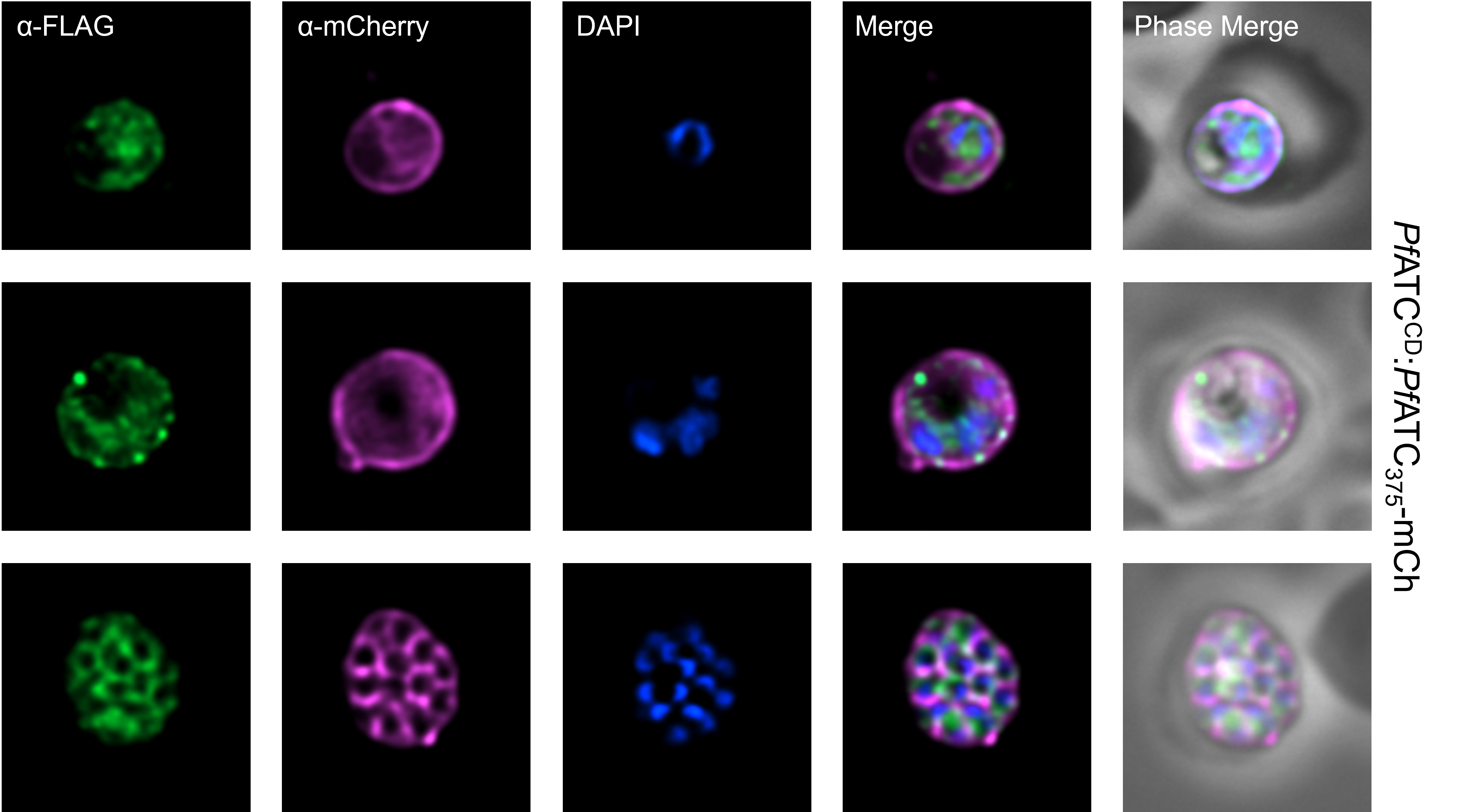

Supplement: S5 Fig — Representative IFA images of PfATCCD:PfATC375-mCh parasites probed with α-FLAG (green) and α-mCherry (magenta) antibodies for detection of endogenous PfATC-2× FLAG and second-copy PfATC-mCherry, respectively. DAPI (blue) stains the parasite nucleus. Images represent fields that are 10 μm long by 10 μm wide. (TIF) [file ppat.1014269.s005.tif]

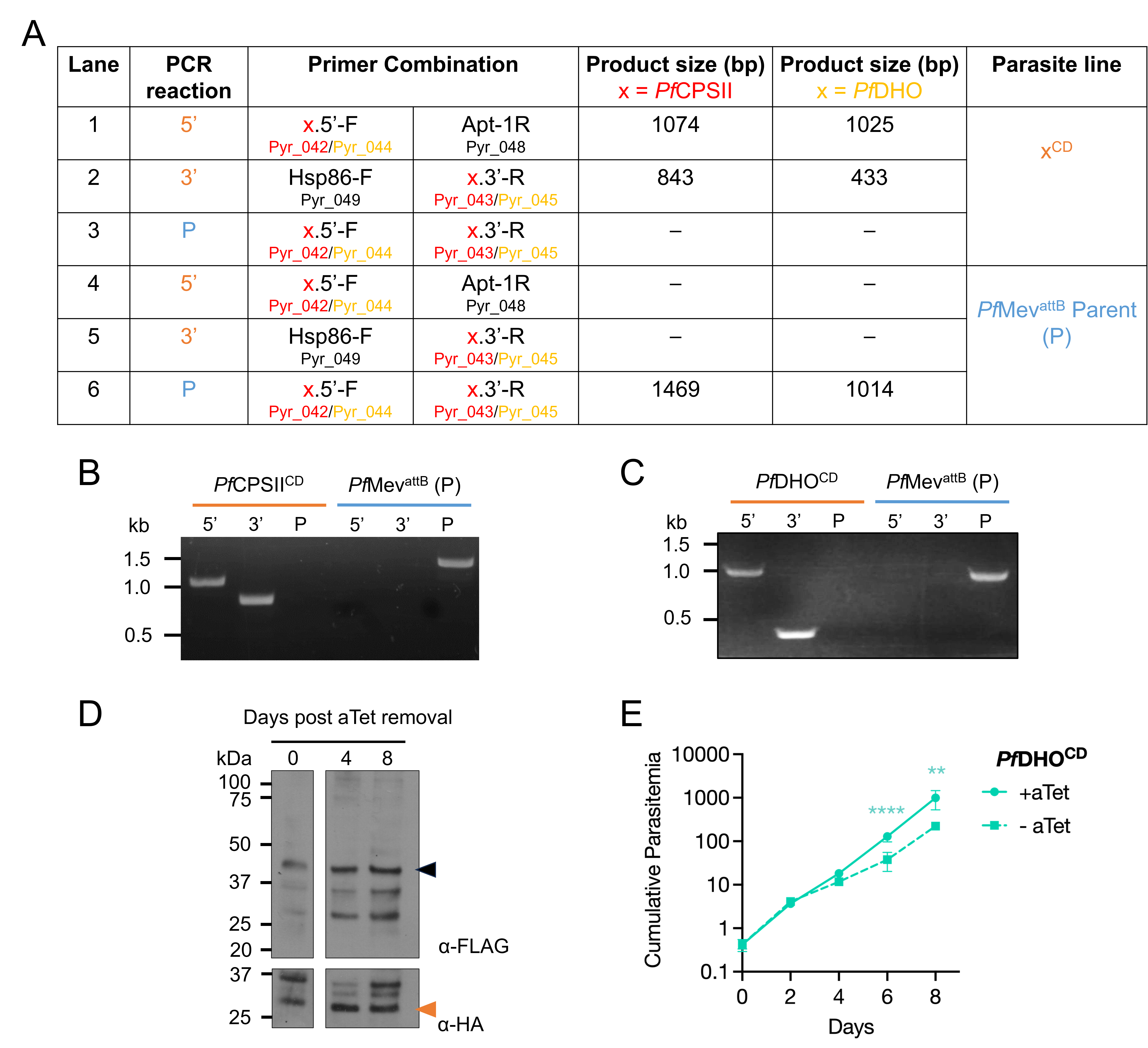

Supplement: S6 Fig — (A) Primer pairs for verifying integration of the pKD-PfCPSII plasmid and pTDN-PfDHO plasmid into the 3’ UTR of PfCPSII and PfDHO genes, respectively, are listed along with the expected sizes of the PCR products. (B) Integration of the pKD-PfCPSII plasmid, and (C) the pTDN-PfDHO plasmid to create PfCPSIICD and PfDHOCD parasite lines, respectively, was validated by PCR amplification of the 5’ and 3’ loci at the genomic insertion sites (orange). The PfMevattB parent (P) line served as a control (blue). Refer to S3A Fig for a schematic of knockdown plasmid insertion into the 3’ UTR of target genes. (D) Western blot analysis of PfDHOCD parasites at Day 0, 4 and 8 of aTet withdrawal, using α-FLAG antibody, showed no measurable knockdown of FLAG-tagged PfDHO (black arrowhead). The α-HA antibody detecting HA-tagged api-SFG (orange arrowhead) was used as a loading control. (E) Growth of PfDHOCD parasites in the presence or the absence of aTet was monitored by flow cytometry for 8 days. Removal of aTet led to a reduction in parasite growth. The means of technical replicates from each experiment were used for plotting and statistical analysis using GraphPad Prism 10 (GraphPad Software, Inc). Cumulative parasitemia was plotted on a log-scale Y-axis with standard deviation. Data were analyzed using two‑way ANOVA, followed by Bonferroni’s multiple‑comparison correction (**, P ≤ 0.01; ***, P ≤ 0.001; ****, P ≤ 0.0001). (TIF) [file ppat.1014269.s006.tif]

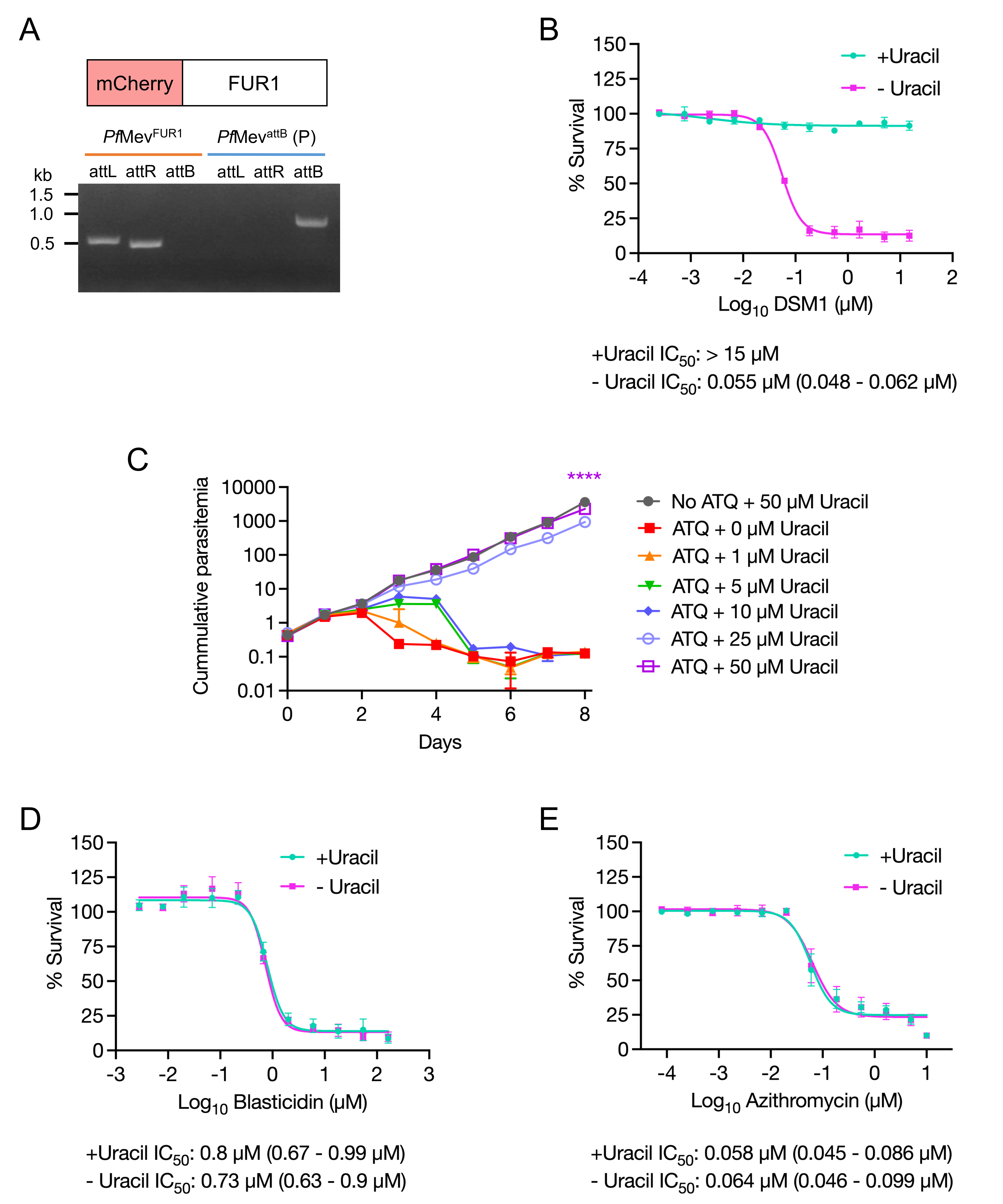

Supplement: S7 Fig — (A) Genomic integration of p15.mCherry-FUR1 in PfMevattB (Parent, ‘P’) parasites was confirmed by PCR amplification (attL and attR products). The intact P230p locus (attB product) was detected only in the parent (blue) and not in the transgenic line (orange). Refer to S1A Fig for a detailed illustration of the integration of p15.mCherry plasmids into attB sites in the P. falciparum genome. Primer pairs for verifying integration of the p15.Cherry plasmid into the P230p locus are listed in S1B Fig along with the expected sizes of the PCR products. (B) PfMevFUR1 parasites ± uracil were exposed to a range of DSM1 concentrations for 72 h, after which parasitemia was quantified by flow cytometry. Data represent the means of three independent biological experiments with quadruplicate samples; error bars indicate standard deviations. Calculated IC₅₀ values with 95% confidence intervals are shown below the graph. (C) PfMevFUR1 parasites were treated with either 0 or 500 nM atovaquone (ATQ) in combination with increasing concentrations of uracil. Parasitemia was quantified daily by flow cytometry, with 1:10 dilutions performed every two days to prevent overgrowth. The means of technical replicates from each experiment were used for plotting and statistical analysis using GraphPad Prism 10 (GraphPad Software, Inc). Cumulative parasitemia was plotted on a log-scale Y-axis with standard deviation. Data were analyzed using two‑way ANOVA, followed by Bonferroni’s multiple‑comparison correction; ****, P ≤ 0.0001. P-value only shown for multiple comparison analysis between samples from ATQ + 50 μΜ Uracil and No ATQ + 50 μM control conditions. The addition of 50 μM uracil largely restored growth to parasites treated with ATQ. (D-E) PfMevFUR1 parasites cultured with or without uracil were exposed to various concentrations of blasticidin-S (D) or azithromycin (E) for 72 or 96 h, respectively. This was followed by measurement of parasitemia via flow cytometry. Data reflect the means of [file ppat.1014269.s007.tif]

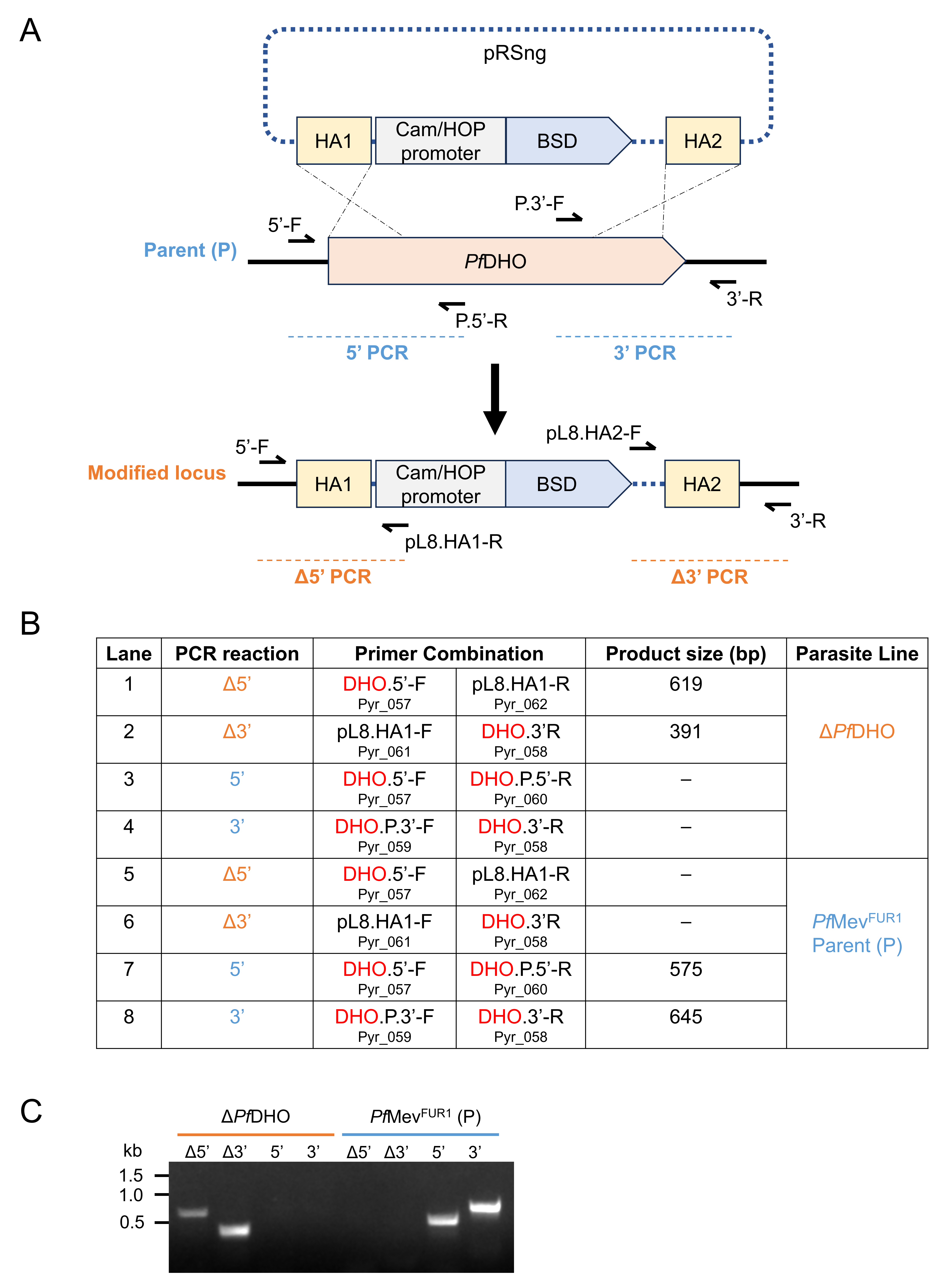

Supplement: S8 Fig — (A) The pRSng plasmid encodes a blasticidin deaminase (BSD) expression cassette flanked by sequences with homology to PfDHO, indicated by dotted lines. The Cam/HOP promoter drives expression of BSD. Arrows indicate the positions of primers used for diagnostic PCR. HA1 and HA2 are the homology arms utilized for recombination of pRSng into the PfDHO locus in PfMevFUR1 parasites (Parent, ‘P’). (B) Primer pairs for verifying gene knockouts are listed along with the expected sizes of the PCR products. (C) The disruption of PfDHO was confirmed by PCR amplification (Δ5’ and Δ3’ products). The intact PfDHO locus (5’ and 3’ products) was detected only in the parent (blue) and not in the ΔPfDHO line (orange). (TIF) [file ppat.1014269.s008.tif]

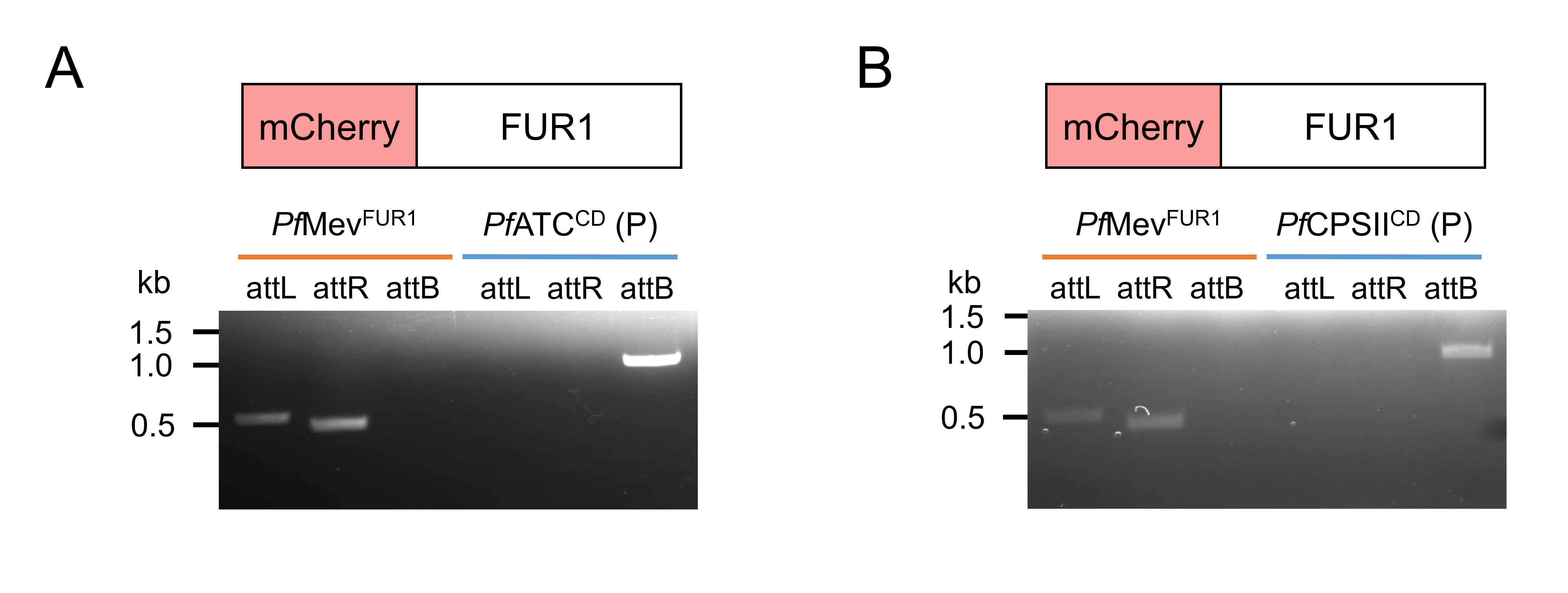

Supplement: S9 Fig — Genomic integration of the p15.mCherry-FUR1 plasmid at the P230p locus in (A) PfATCCD (Parent, ‘P’) or (B) PfCPSIICD (Parent, ‘P’) parasites was confirmed by PCR amplification of attL and attR products. The intact P230p locus (attB product) was detected only in the parent (blue) and not in the transgenic lines (orange). For a detailed schematic of p15.mCherry plasmid integration into attB sites, refer to S1A Fig. Primer pairs for verifying integration into the P230p locus are listed in S1B Fig along with the expected PCR product sizes. (TIF) [file ppat.1014269.s009.tif]

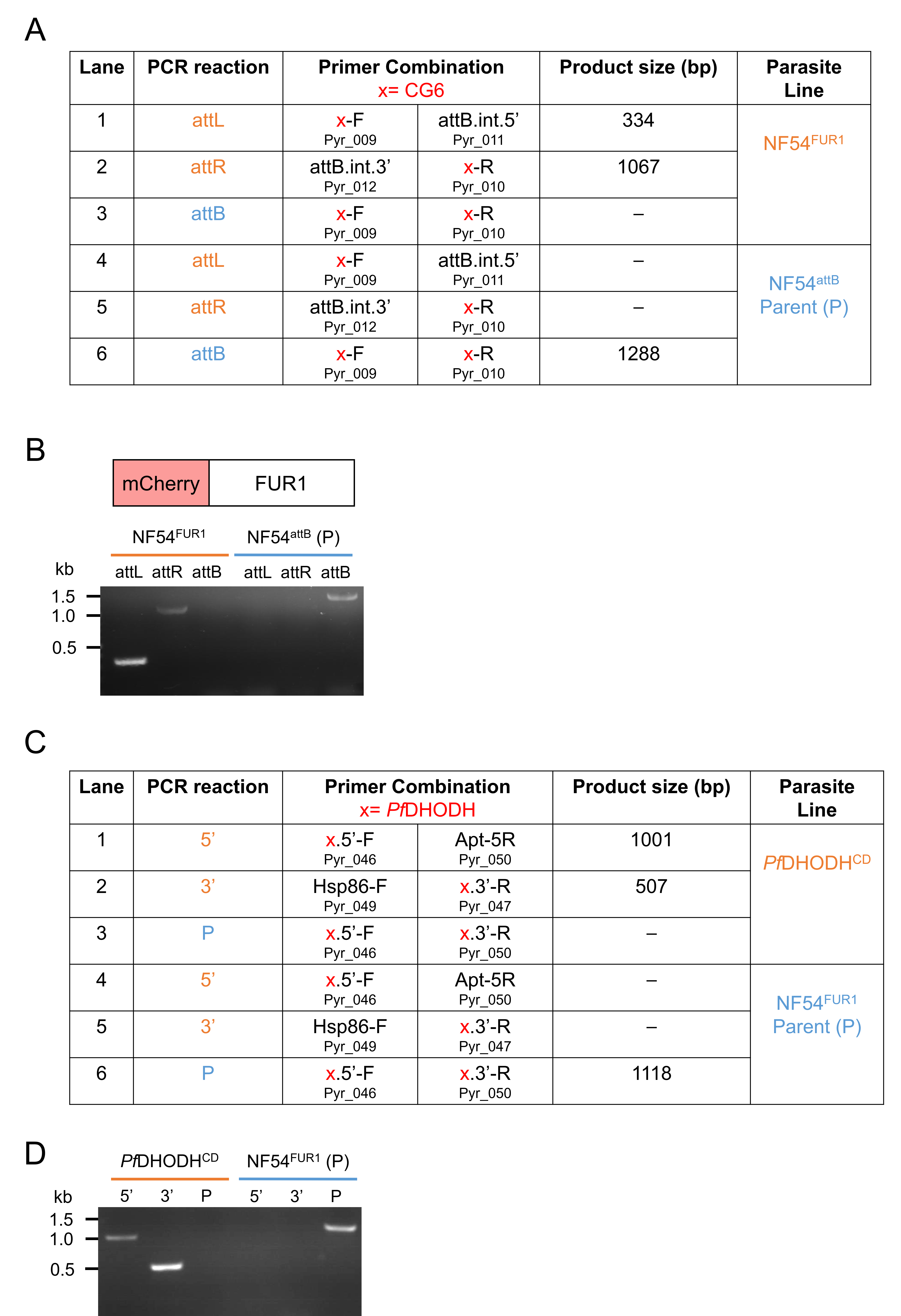

Supplement: S10 Fig — (A) Primer pairs for verifying p15.Cherry-FUR1 integration into the CG6 genomic locus of NF54attB parasites (Parent, ‘P’) are provided. (B) Genomic integration at the CG6 locus was confirmed by PCR (attL and attR products), with the intact CG6 locus (attB product) detected only in the parent line (blue) and not in the transgenic line (orange). Refer to S1A Fig for a detailed schematic of p15.mCherry plasmid integration into attB sites. (C) Primer pairs for verifying integration of the pKDtagless-PfDHODH plasmid into the 3’ UTR of PfDHODH are listed. (D) The genotype of the PDHODHCD parasite line was confirmed by PCR amplification of the 5’ and 3’ loci at the genomic insertion site (orange). The NF54FUR1 parent (P) line served as a control (blue). Refer to S3A Fig for a schematic of knockdown plasmid insertion into the 3’ UTR of target genes. (TIF) [file ppat.1014269.s010.tif]

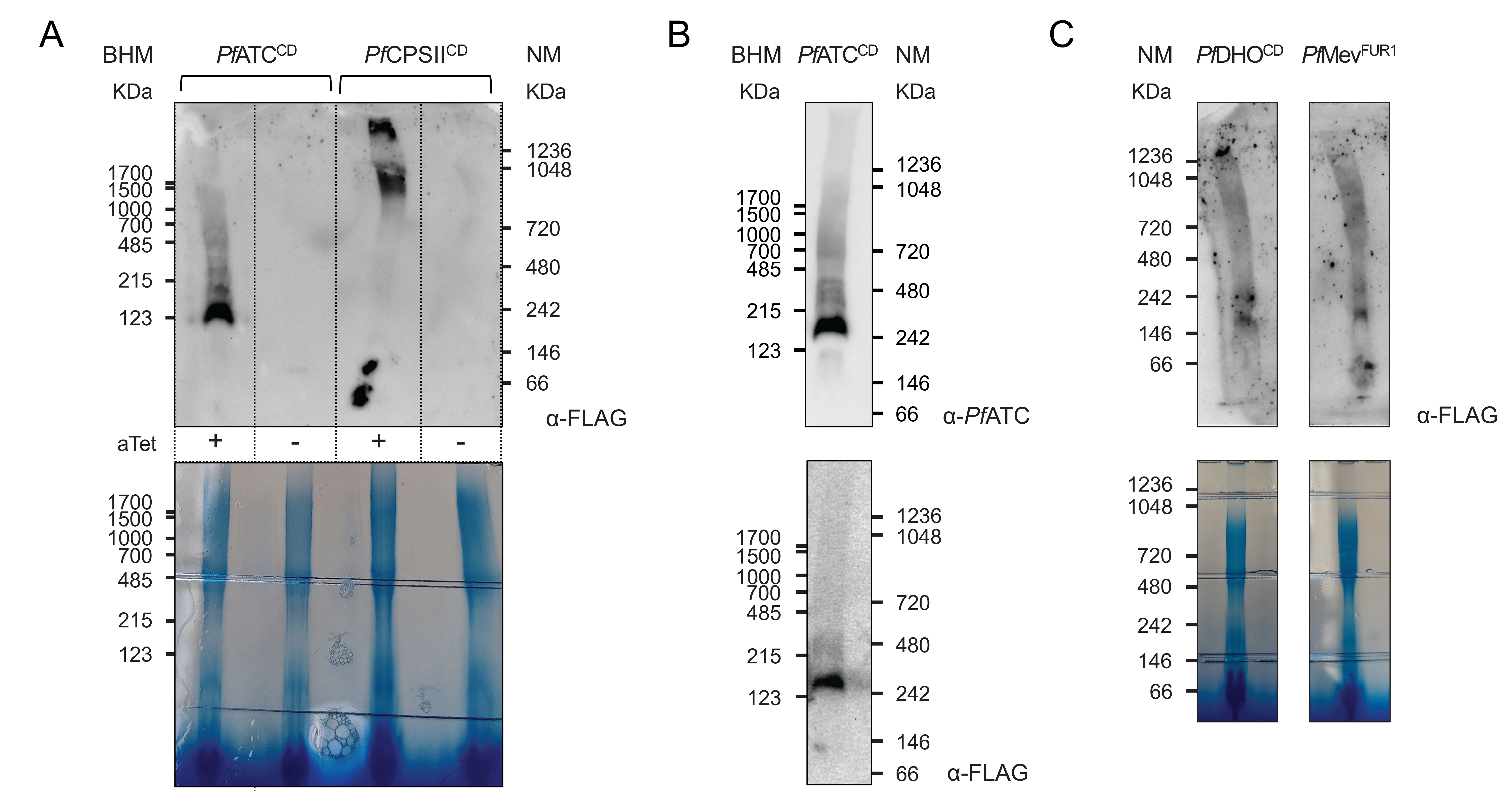

Supplement: S11 Fig — (A) α-FLAG immunoblot (top) and the corresponding BN-PAGE gel (bottom) of PfATCCD and PfCPSIICD lysates from parasites cultured ± aTet. Several bands seen in +aTet conditions were not detected in - aTet samples. (B) Immunoblot of PfATCCD + aTet sample separated by BN-PAGE, probed with α- PfATC (top) and then stripped and reprobed with α-FLAG (bottom) antibodies, revealed a prominent band along with several fainter higher molecular weight species. (C) α-FLAG immunoblot (top) of PfDHOCD and PfMevFUR1 (parent) lysates did not detect any specific band corresponding to PfDHO-3× FLAG. The BN-PAGE gel (bottom) demonstrates comparable protein loading. Bovine heart mitochondria (BHM) and the NativeMark Unstained Protein Standard (NM) served as native molecular mass references in panels (A) and (B), whereas panel (C) included only NM. The two ladders migrate differently on BN-PAGE, likely due to differences in protein composition and Coomassie dye binding under native conditions. Note: Apparent lane shifts between immunoblots and the BN-PAGE gel are due to minor gel misalignment during transfer. (TIF) [file ppat.1014269.s011.tif]

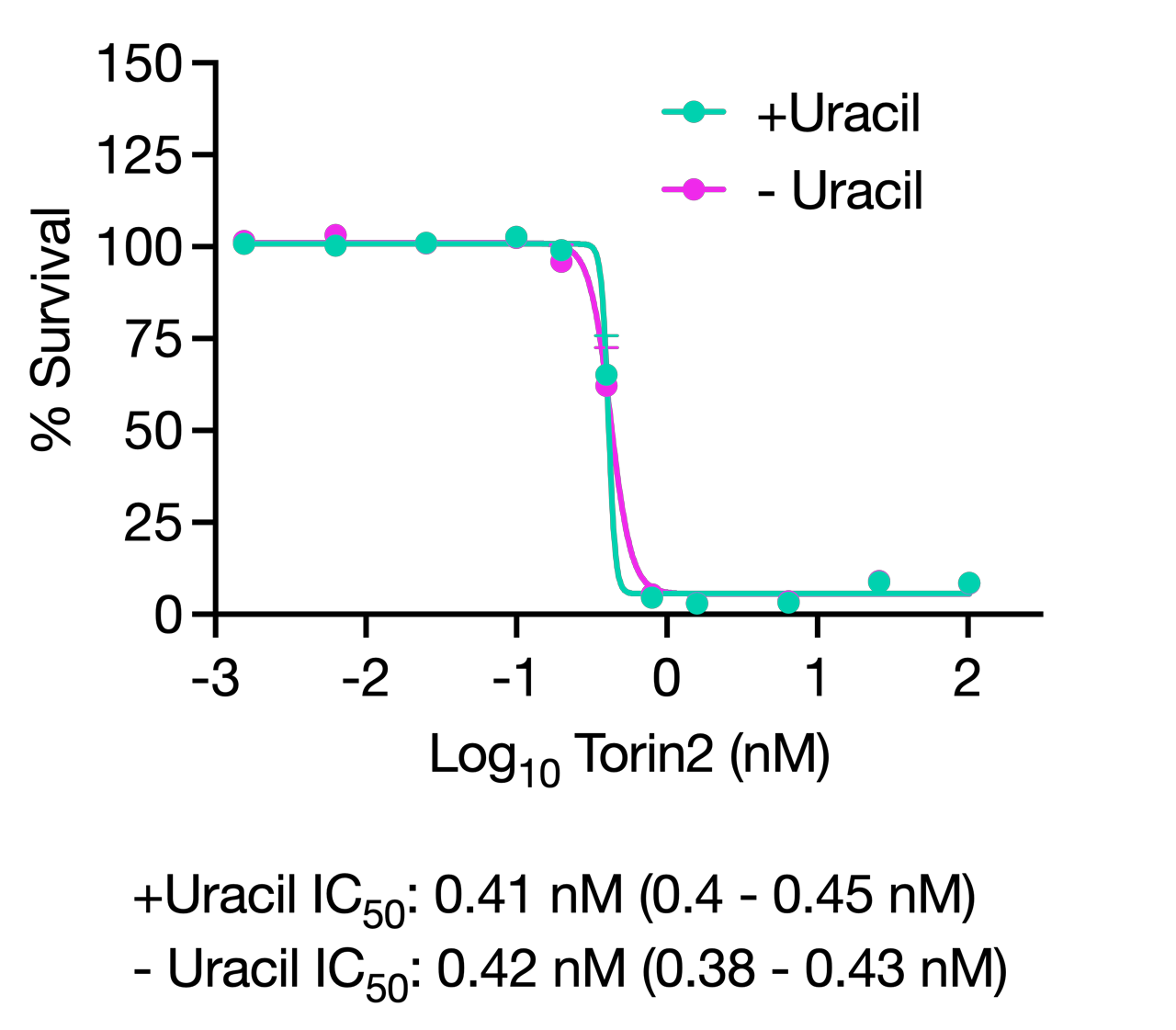

Supplement: S12 Fig — PfATCCD parasites expressing FUR1 were exposed to a range of Torin 2 concentrations with or without 50 μM uracil for 72 h, after which parasitemia was quantified by flow cytometry. Data represent the means of three independent biological experiments with quadruplicate samples; error bars indicate standard deviations. Calculated IC₅₀ values with 95% confidence intervals are shown below the graph. (TIFF) [file ppat.1014269.s012.tiff]

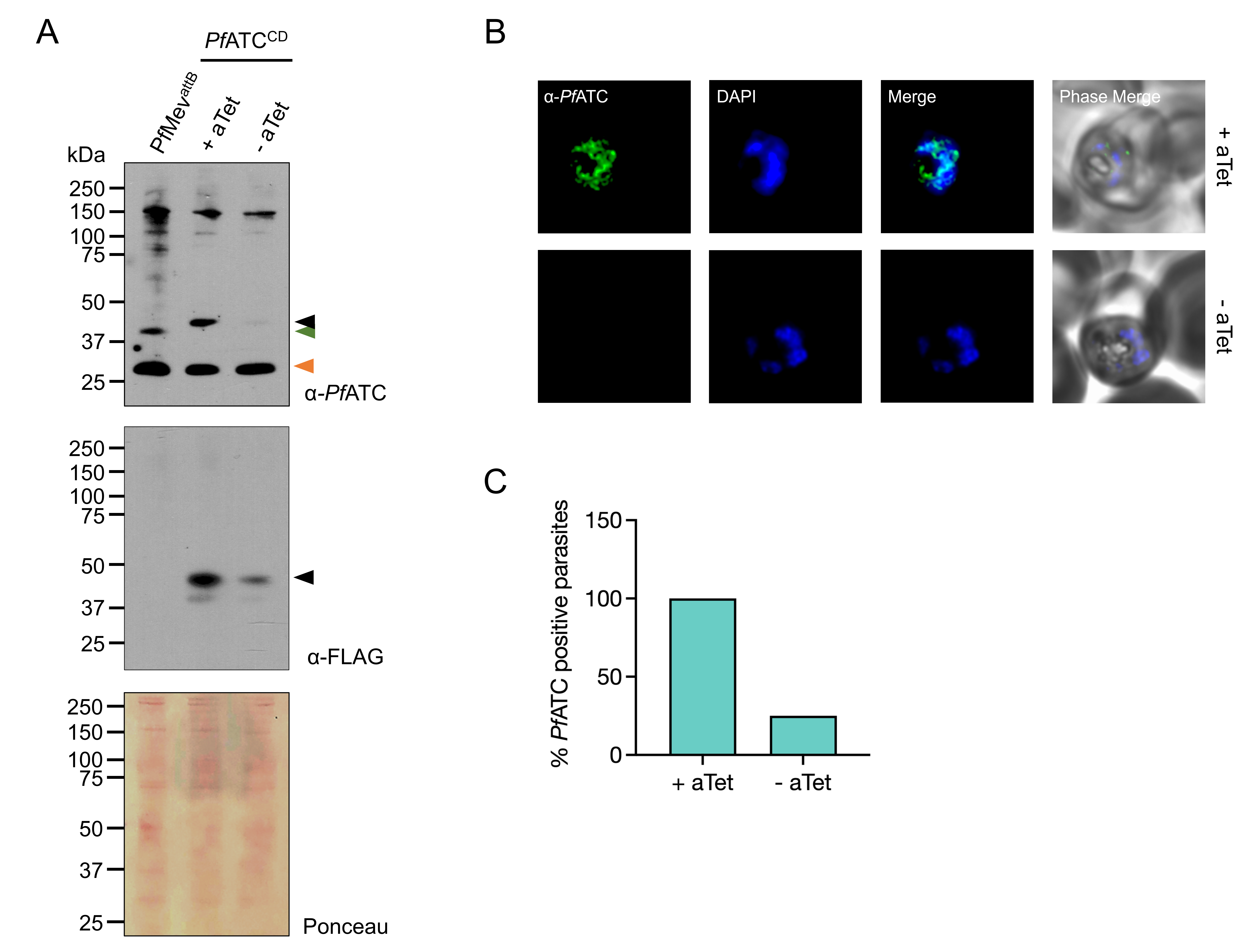

Supplement: S13 Fig — (A) Lysates from PfMevattB (control) and PfATCCD parasites were probed with α-PfATC. The antibody detected a ~ 40 kDa band in PfMevattB lysates (green arrow), consistent with the predicted molecular weight of PfATC (43.3 kDa). In PfATCCD parasites, a slightly higher band was observed (black arrow, +aTet condition) but absent when PfATC was depleted by removal of aTet from culture media (-aTet). This higher product is consistent with the predicted 45 kDa size of the endogenously C-terminally 2× FLAG tagged PfATC. The orange arrow marks a non-specific band. The blot was stripped and reprobed with an α-FLAG antibody, which detected the same band in the + aTet PfATCCD sample that was lost upon aTet withdrawal. Ponceau S staining is shown to indicate relative protein loading. (B) PfATCCD parasites cultured under +aTet and -aTet conditions were fixed and probed with affinity-purified α-PfATC in an immunofluorescence assay. Representative images show specific staining by α-PfATC antibodies (green) in parasites grown under +aTet conditions, while no signal was detected in parasites grown under -aTet conditions. Images represent fields that are 10 μm long by 10 μm wide. (C) Fraction of PfATCCD parasites exhibiting α-PfATC staining under +aTet and -aTet conditions. All parasites in the +aTet condition were positive for α-PfATC staining (total cells imaged = 16), while the signal was markedly reduced in the -aTet condition (total cells imaged = 36). (TIF) [file ppat.1014269.s013.tif]
